# Spatial Regulation of Acceptor Units in Olefin‐Linked COFs toward Highly Efficient Photocatalytic H_2_ Evolution

**DOI:** 10.1002/advs.202203832

**Published:** 2022-08-18

**Authors:** Zhengfeng Zhao, Xuepeng Chen, BaoYing Li, Shu Zhao, Liwei Niu, Zhenjie Zhang, Yao Chen

**Affiliations:** ^1^ School of Chemistry and Chemical Engineering Qilu University of Technology (Shandong Academy of Sciences) Jinan 250353 P. R. China; ^2^ State Key Laboratory of Medicinal Chemical Biology College of Pharmacy Nankai University Tianjin 300071 P. R. China; ^3^ Institute of Advanced Battery Materials and Devices Faculty of Materials and Manufacturing Beijing University of Technology Beijing 100124 P. R. China

**Keywords:** carrier diffusion length, olefin‐linked covalent organic frameworks (COFs), photocatalytic hydrogen production, Pt nanoparticles, spatial distance

## Abstract

Covalent organic frameworks (COFs)‐based photocatalysts have received growing attention for photocatalytic hydrogen (H_2_) production. One of the big challenges in the field is to find ways to promote energy/electron transfer and exciton dissociation. Addressing this challenge, herein, a series of olefin‐linked 2D COFs is fabricated with high crystallinity, porosity, and robustness using a melt polymerization method without adding volatile organic solvents. It is found that regulation of the spatial distances between the acceptor units (triazine and 2, 2'‐bipyridine) of COFs to match the charge carrier diffusion length can dramatically promote the exciton dissociation, hence leading to outstanding photocatalytic H_2_ evolution performance. The COF with the appropriate acceptor distance achieves exceptional photocatalytic H_2_ evolution with an apparent quantum yield of 56.2% at 475 nm, the second highest value among all COF photocatalysts and 70 times higher than the well‐studied polymer carbon nitride. Various experimental and computation studies are then conducted to in‐depth unveil the mechanism behind the enhanced performance. This study will provide important guidance for the design of highly efficient organic semiconductor photocatalysts.

## Graphical abstract

The spatial regulation of acceptor units in olefin‐linked COFs to match the charge carrier diffusion length can dramatically promote the exciton dissociation, hence leading to outstanding photocatalytic H_2_ evolution performance. This study endows COFs with great potential to serve as excellent platforms for solar energy conversion to H_2_ with an apparent quantum yield of 56.2% at 475 nm.

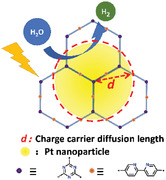

## 1 Introduction

**Figure 1:**
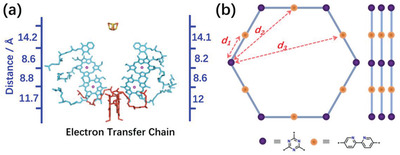
Illustration of inter active centers distances in COFs. a) The inter distance active centers distance (electron acceptors) in natural electron transfer chain^[^ ^2^ ^]^; the ball in porphyrin units: coordinates Mg^2+^. b) Precisely control the distance between Bpy and TA units by tuning the size of the monomers in COFs.

Photocatalytic hydrogen (H_2_) evolution represents a promising technology for converting solar energy to fuel.^[^ ^1^ ^]^ The main challenge is to ensure the efficient dissociation of the photogenerated exciton for improving photocatalytic performance. In photosynthetic proteins (PS I), the light utilization is governed by the extremely distance‐dependent energy/electron transfer between the active centers (electron acceptors) in the electron transfer chain.^[^ ^2^ ^]^ Typical center‐to‐center distances of neighboring electron acceptors are consequently close with slightly different corresponding to the diffusion and dissociation of the excited states, so energy/electron‐transfer rates are fast (**Figure** **1**a).^[^ ^3^ ^]^ Previous research has shown that the energy/electron transfer^[^ ^4^ ^]^ and the inter active centers’ distance^[^ ^5^ ^]^ are the key factors for the overall catalytic performance. Hence, one proposed solution to exciton dissociation is the introduction of different electron acceptors as active centers into the artificial photocatalytic system for the energy/electron transfer process. Simultaneously, optimizing the distances of neighboring active centers matches the diffusion length of excited states for boosting the dissociation of the photogenerated exciton.

Covalent organic frameworks (COFs),^[^ ^6^ ^]^ as a typical class of crystalline porous framework polymers, have attracted increasing attention as photocatalytic candidates due to their highly conjugated, adjustable structures, and anisotropic transport of photogenerated charges.^[^ ^7^ ^]^ COFs are usually synthesized by condensation of different monomers, which greatly facilitates the ordered assembly and spatial distance regulation of various acceptor units. Another distinct feature of COFs is their adjustable distance of active centers, which can be controlled by the geometry and size of the monomers. All above natures are difficult to be achieved by other inorganic or organic photocatalysts. It should be noted that the physical and chemical properties of COFs depend on the bridging bond linkages.^[^ ^8^ ^]^ Compared with the reversible chemical bond bridged configurations (boronate, boroxine, imine, azine, and hydrazone),^[^ ^6b^ ^]^ olefin‐linked COFs are the competitive choice for photocatalytic applications in aqueous systems due to their extended conjugated structures and high chemical stability.^[^ ^9^ ^]^ In particular, the extended *π*‐conjugated configurations of olefin‐linked COFs form the densely aligned *π* columns, which serve as pre‐organized pathways to facilitate photoexcited charge‐carrier transport.^[^ ^10^ ^]^

In the past decades, many strategies have been developed to boost the photocatalytic performance of COFs, such as active molecular building blocks stack,^[^ ^11^ ^]^ donor–acceptor combination,^[^ ^12^ ^]^ crystallinity improvement,^[^ ^7^, ^13^ ^]^ heterojunction construction.^[^ ^14^ ^]^ Although the photocatalytic efficiency of COFs has been significantly improved, their light conversion efficiency (apparent quantum yield, AQY) of photocatalytic H_2_ evolution still has great room for improvement (only ≈10% at present, Table S1, Supporting Information). Photogenerated intrinsic Frenkel type excitons of COFs possess large binding energy^[^ ^1a^ ^]^ and short diffusion length (≈5 nm),^[^ ^15^ ^]^ which is the main factor limiting its photocatalytic performance. To further enhance photocatalytic H_2_ evolution, developing new COF platforms with matched spatial distances of electron acceptors for the fast energy/electron transfer to promote Frenkel exciton dissociation and suppress electron–hole recombination is highly desired but remains challenging.

Herein, addressing this challenge, we built an olefin‐linked COF platform installed with 2, 2'‐bipyridine (Bpy) and 1,3,5‐ triazine (TA) units as the essential acceptors to impel the energy/electron transfer in COF frameworks for photocatalytic H_2_ evolution (Figure 1b). This COF platform (NKCOF‐112‐M, ‐113‐M, and ‐114‐M, NKCOF = Nankai Covalent Organic Framework, M = Melt polymerization method) with the same acceptors (Bpy and TA units) allowed us to correlate the spatial distance between the acceptors with the photocatalytic performance of these COFs. Finally, we achieved a high AQY of 56% at 475 nm for photocatalytic H_2_ evolution at room temperature, only lower than CYANO‐CON^[^ ^16^ ^]^ among all COF‐based photocatalysts. Notably, photocatalytic overall water splitting was found to proceed by Pt modified NKCOF‐113‐M, which was rarely achieved by pure COF photocatalyst (The unique example is Covalent Triazine Framework (CTF)^[^ ^13^, ^17^ ^]^). We then studied the photodeposition behaviors of Pt nanoparticles (the co‐catalyst for H_2_ evolution) on COFs and charge carrier diffusion length (*L*_D_) to unveil the mechanism. Thus, a fundamental understanding of inter acceptor distance on photocatalyst will provide important guidance for the design of highly efficient photocatalytic systems for H_2_ evolution.

## 2 Results and Discussion

### 2.1 Design and Synthesis of Olefin‐Linked COF Photocatalysts

**Figure 2:**
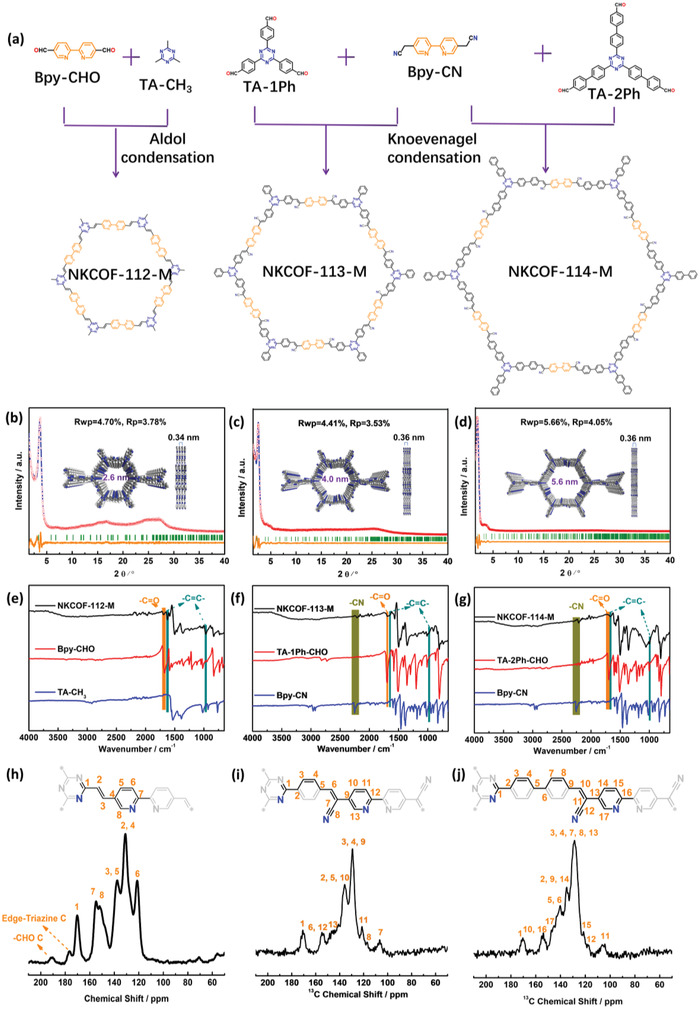
a) Chemical structure of the COFs; experimentally observed PXRD patterns (red cycle), Pawley‐refined patterns (blue line), Bragg positions (green line), the difference between the experimental (orange line), and calculated patterns of b) NKCOF‐112‐M, c) NKCOF‐113‐M, and d) NKCOF‐114‐M for AA stacking; FT‐IR spectra of COFs and the corresponding monomers, e) NKCOF‐112‐M, f) NKCOF‐113‐M, and g) NKCOF‐114‐M; solid‐state ^13^C CP/MAS NMR spectra of h) NKCOF‐112‐M, i) NKCOF‐113‐M, and j) NKCOF‐114‐M.

It was well known that the Bpy^[^ ^18^ ^]^ and TA‐based^[^ ^19^ ^]^ materials have been widely used in photocatalysis. Besides, the strong coordinating ability of the Bpy unit with transition metals impels the construction of reduction sites^[^ ^20^ ^]^ in COFs. We then chose Bpy and TA‐based monomers as core building blocks to construct the olefin‐linked COFs. It should be noted that the spatial distance between the acceptor units was controlled by the number of phenylene between Bpy and TA units. The geometric relationship of spatial distances between adjacent acceptor units in the plane is shown in Figure 1b. These olefin‐linked COFs were synthesized using a melt polymerization method developed by our group.^[^ ^21^ ^]^ The major advantages of the melt polymerization method lie in: 1) volatile organic solvents are not used. Instead, fluxing mediums such as benzoic anhydride with low melting and high boiling points are used. 2) The method can facilitate scale‐up fabrication of COFs. Here, we found that the melt polymerization method using benzoic anhydride as a reaction medium worked for not only Aldol condensation (NKCOF‐112‐M), but also Knoevenagel condensation (NKCOF‐113‐M and NKCOF‐114‐M) (**Figure** **2**a). After synthesis reaction and simple purification, the intense and narrow peaks in the powder X‐ray diffraction (PXRD) patterns (Figure 2b–d) revealed the high crystallinity of the COFs. We combined experimental PXRD patterns with the structural simulation to determine their crystallinity. The fully eclipsed AA layer stacking and staggered AB layer stacking models were built using the Materials Studio software package (Figure S1, Supporting Information). Pawley refinements of the observed PXRD profiles confirmed the assignment of observed reflections. All the experimentally observed PXRD patterns matched well with the eclipsed AA model, as evidenced by the small differences and low *R*_wp_ and *R*_p_ values. The sharp PXRD peaks of NKCOF‐112‐M, NKCOF‐113‐M, and NKCOF‐114‐M could be assigned to the (100) facet of a highly regular lattice that was stacked in a hexagonal orientation. The unit cell parameters with the *P‐6* space group and the coordinates of the refined AA stacking models corresponding to the COFs are provided in Tables S2–S4 (Supporting Information).

The chemical structures of the COFs were characterized by Fourier transform infrared (FT‐IR) spectra and solid‐state ^13^C cross‐polarization magic angle spinning nuclear magnetic resonance (CP/MAS NMR) spectra. In FT‐IR spectra, the newly formed signal peaks at 1622 and 990 cm^−1^ are assigned to the stretching vibration of trans —C=C— linkages (Figure 2e–g). The significantly weakened C=O stretching band (1690 cm^−1^) manifested the high polymerization degrees for the COFs. In addition, the intense peaks of —CN at ≈2220 cm^–1^ on the FT‐IR spectra of NKCOF‐113‐M and NKCOF‐114‐M revealed the successful Knoevenagel condensation by benzoic anhydride. In solid‐state ^13^C CP/MAS NMR spectra of the COFs, the characteristic signals of the carbons can be assigned to their corresponding chemical structures (Figure 2h–j). The slight residual carbonyl (—C=O) signal located at *δ*≈190 ppm and edge‐1, 3, 5‐triazine (partial methyl groups participate in the construction of the framework) signal located at *δ*≈175 ppm were observed from the NKCOF‐112‐M sample. The signals at ≈130 and ≈138 ppm were assigned to the olefin linkages. The strong resonances at 170 ppm originated from the TA units in the framework. In contrast, the emerged resonance at ≈105 ppm was assigned to the cyano carbons in NKCOF‐113‐M and NKCOF‐114‐M samples. The signals of —C=C— were shifted to ≈105 and ≈135 ppm due to the cyano effect. Besides, the signals of —C=O can hardly be detected, suggesting a high degree of polymerization.

The chemical stability was evaluated by immersing the three COFs in boiling water, aqueous NaOH (10 m), and HCl (12 m) solutions for 24 h (Figure S2, Supporting Information). The diffraction peak intensity of the boiling water and NaOH‐treated sample was consistent with that of the original sample. In aqueous HCl, the sharp PXRD peaks disappeared or were weakened due to the lattice distortion caused by N‐site ionization^[^ ^22^ ^]^ (Figure S2d, Supporting Information). After treatment with 1 m NaOH for 1 h, the crystallinity of HCl‐treated samples was fully recovered (the blue curves in Figure S2a—c, Supporting Information). The FT‐IR spectra of the treated COFs in the harsh conditions showed no significant difference (Figure S3, Supporting Information). These results indicated the high chemical stability of olefin‐linked COFs against harsh conditions.

**Figure 3:**
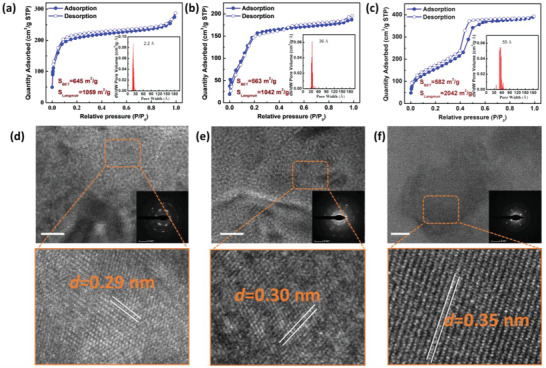
N_2_ sorption isotherms and pore size distribution curves of a) NKCOF‐112‐M, b) NKCOF‐113‐M, and c) NKCOF‐114‐M; HRTEM and the corresponding SAED images of d) NKCOF‐112‐M, e) NKCOF‐113‐M, and f) NKCOF‐114‐M, scale: 10 nm.

The synthesized COFs are activated in supercritical CO_2_ after washing with methanol and tetrahydrofuran in a Soxhlet extractor for 48 h. To investigate the intrinsic porosity of the COFs, N_2_ adsorption isotherms were performed at 77 K. The N_2_ adsorption–desorption isotherms of NKCOF‐112‐M and NKCOF‐113‐M showed the typical type‐II isotherm, which indicates the presence of mesopores (**Figure** **3**a). The Brunauer−Emmett−Teller (BET) and Langmuir surface areas were calculated to be 645 and 1059 m^2^ g^−1^ for NKCOF‐112‐M. The pore size distribution (PSD) using the density functional theory (DFT) model revealed that the pore size of NKCOF‐112‐M centered at 2.2 nm. The BET and Langmuir surface areas were calculated to be 563 and 1042 m^2^ g^−1^ for NKCOF‐113‐M (Figure 3b). The PSD calculated by the DFT method gives rise to the pore size centered at 3.6 nm. NKCOF‐114‐M exhibits typical type IV isotherms (Figure 3c), suggesting the relatively large mesopore. The BET and Langmuir specific surface areas were determined to be 582 and 2042 m^2^ g^−1^. The PSD profile of NKCOF‐114‐M exhibited a pore width of 5.6 nm, the largest pore size among all reported olefin‐linked COFs (Table S5, Supporting Information). Note that the PSD of all three COFs agrees with the predicted AA eclipsed stacking models. High‐resolution transmission electron microscope (HRTEM) images and the selected area electron diffraction (SAED) patterns (Figure 3d,e) were also taken further to gain insights into the crystal structure of the COFs. In the enlarged regions from the HRTEM images, all three COFs show similar lattice fringes of ≈0.3 nm, corresponding to the layer spacing of *π*–*π* stacking. Furthermore, the bright diffraction spots and rings in the related SAED patterns (the insets) also verified the high crystalline structures of the COFs.

### 2.2 Optical and Electronic Properties of the COFs

**Figure 4:**
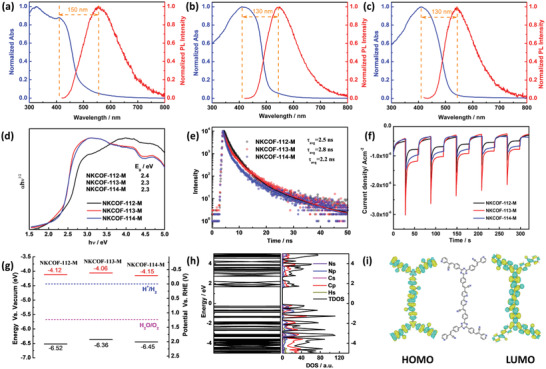
Normalized UV–vis diffuse reflectance spectra (DRS) and steady‐state photoluminescence (PL) spectra excited at 400 nm of a) NKCOF‐112‐M, b) NKCOF‐113‐M, and c) NKCOF‐114‐M; d) *E*_g_ of all COFs determined from Kubelka–Munk‐transformed DRS; e) excited state decay spectra of the COFs (Photoexcitation at 400 nm); f) photocurrent generation of the COFs upon light (*λ* ≥ 420 nm) on/off switch, which was measured by coating the materials on the FTO as a working electrode in a three‐electrode CV setup; g) the band edge positions of the COFs (the Vacuum‐level values are converted to electrochemical potentials as follows: −4.44 eV versus vacuum level is equal to 0 V versus reversible hydrogen electrode (RHE) at pH 7; *E*_CB_ = *E*_VB_+*E*_g_); h) electronic properties of NKCOF‐113‐M using DFT calculations and corresponding density of states (DOS). i) DFT optimized molecular orbital plots of NKCOF‐113‐M.

For the photocatalytic application of the COFs, we systematically studied the optical and electronic properties. UV–vis diffuse reflectance spectra (UV–vis DRS) and steady‐state photoluminescence (PL) spectra clarified the ground and excited state features of the COFs (**Figure** **4**a–c). UV–vis DRS showed that the COFs could absorb light from the UV region to the visible region with an absorption edge of 515 nm for NKCOF‐112‐M, and 540 nm for NKCOF‐113‐M and NKCOF‐114‐M. The light‐harvesting capacity of COFs can be slightly improved by increasing the spatial distance between Bpy and TA units. However, the longer distance between acceptors had little effect on the light‐harvesting capacity of COFs, mainly because the rotational single bonds between benzene rings contribute less to the *π*‐conjugation. In addition, steady‐state PL of all samples excited above the band edge (420 nm). Their fluorescence characteristics mainly resulted from the extended *π*‐conjugated and aggregation‐induced emission (AIE) of the dense packing olefin linkage. The PL of the COFs was broad with a large Stokes shifts (Δ*λ* = 150 nm for NKCOF‐112‐M, 130 nm for NKCOF‐113‐M and NKCOF‐114‐M), which can be attributed to the energy loss by charge‐transfer^[^ ^23^ ^]^ and vibrational relaxation. The PL results proved that the longer distance between acceptors narrows the Stokes shift, which is beneficial to the efficient conversion of solar energy. Furthermore, the optical band gaps (*E*_g_) were calculated as shown in Figure 4d according to the Kubelka–Munk function from UV–vis DRS.

The PL decay lifetimes of the COFs were measured in the solid‐state (Figure 4e). The COFs exhibited fast (*τ*_1_) and slow (*τ*_2_ and *τ*_3_) excited‐state lifetime, as determined from triple exponential function fitting (Table S6, Supporting Information). The rather different lifetimes resulted from the surface trap states (*τ*_1_) and the bulk energy/electron transfer (*τ*_2_) contributions.^[^ ^24^ ^]^ As for the slowest *τ*_3_ may be brought about the energy/electron transfer along with the *π*–*π* stacking. The average decay lifetimes of NKCOF‐112‐M, NKCOF‐113‐M, and NKCOF‐114‐M were estimated to be 2.5, 2.8, and 2.2 ns. Therefore, the matched spatial distance between Bpy and TA units is conducive to the excitons dissociation of the COFs by prolonging their excited‐state lifetime. Exciton dissociation behaviors of the COFs were further investigated by the photoelectrochemical tests. Upon switching the photoirradiation (>420 nm) on and off, the COFs photoelectrodes quickly responded to the incident light and generated photocurrent (Figure 4f). In the long‐term photocurrent response tests for the COFs, the photocurrents were nearly constant within the measurement period, indicating their excellent stability under irradiation. NKCOF‐113‐M possessed a much higher transient photocurrent than other ones corresponding to the most efficient exciton dissociation, which is consistent with the exciton lifetime results.

The electronic structures of the COFs were further investigated by ultraviolet photoelectron spectroscopy (UPS), Mott–Schottky (M–S) measurements, and density‐functional‐theory (DFT) calculations. UPS was used to determine the energy level of valence band maximum (*E*_VB_). The *E*_VB_ of −6.52, −6.36, and −6.45 eV (vs vacuum level) for NKCOF‐112‐M, NKCOF‐113‐M, and NKCOF‐114‐M (Figure S4, Supporting Information) were calculated by subtracting the UPS width from excitation energy (He I, 21.22 eV). The *E*_VB_ of the COFs were more negative than −5.26 eV (vs vacuum level at pH 7, the potential of water oxidation to produce O_2_), suggesting their potential applications for photocatalytic O_2_ evolution. Combined with the *E*_g_, the conduction band minimum (*E*_CB_) could be calculated as −4.12, −4.06, and −4.15 eV versus vacuum level for NKCOF‐112‐M, NKCOF‐113‐M, and NKCOF‐114‐M (Figure 4g). The Mott–Schottky plots (Figure S5, Supporting Information) showed that the three COFs are n‐type semiconductors with more negative flat band potentials (*E*_fb_) than 0 eV (V versus RHE, the potential of water reduction to produce H_2_), suggesting the photocatalytic H_2_ evolution applications. In addition, the most negative *E*_fb_ versus RHE of NKCOF‐113‐M indicated the highest H_2_ evolution driving force.

In addition, we performed DFT calculations to theoretically investigate the electronic structures of NKCOFs. In the projected density of states (PDOS) profile for the COFs (Figure 4h; Figure S6, Supporting Information), the dominant feature is C 2p‐N 2p bonding resonances near the Fermi level and forming hybridized electronic states, which revealed that the top of VB was mainly contributed by the 2p orbital of N and C atoms. Such results suggest that the excited electron on the as‐prepared COFs may arise from the well *π*‐delocalization from the 2p orbital of C and N atoms. The calculated bandgaps (Figure S6c, Supporting Information) of the COFs corresponded well with the experimental values (UV–vis DRS) and confirmed their semiconductive nature. In addition, the charge density distributions of the lowest unoccupied molecular orbital (LUMO) and highest occupied molecular orbital (HOMO) (Figure 4I; Figure S7, Supporting Information) were quite different in the unit cells of the three COFs. Both LUMO and HOMO of NKCOF‐113‐M exhibited stronger electron distribution uniformity than those of the other two COFs as the inter acceptors’ distance changed. It means that the delocalized *π*‐electrons from NKCOF‐113‐M could transfer more easily in the *π*‐conjugate plane.

### 2.3 Photocatalytic H_2_ Evolution

**Figure 5:**
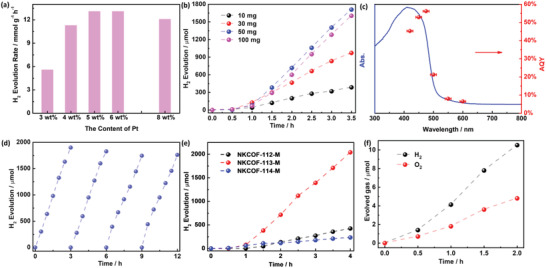
a) Optimization of the Pt wt% loading on the NKCOF‐113‐M surface; b) optimization of Pt@NKCOF‐113‐M quality for HER; c) AQY values of Pt@NKCOF‐113‐M (The error bar came from a bandpass filter); d) long‐term H_2_ evolution test of Pt@NKCOF‐113‐M; e) the photocatalytic H_2_ evolution performance of the three Pt@COFs under the same conditions; f) the photocatalytic overall water splitting activity of Pt@NKCOF‐113‐M.

The surfaces of the NKCOFs can be wetted by water (the hydrophobic angle less than 90° as shown in Figure S8, Supporting Information), which is conducive to the adsorption of water molecules. After confirming the structures and fundamental optical and electrical properties, NKCOF‐113‐M was predicted to possess the best photocatalytic activity among all the three COFs. Consequently, NKCOF‐113‐M was selected to optimize conditions for photocatalytic H_2_ evolution under a Xe lamp (*λ* > 420 nm) using Triethanolamine (TEOA) as a hole‐scavenger. The evolved H_2_ is quantified with the H_2_ standard curve (Figure S9, Supporting Information). In the absence of any co‐catalyst (e.g., Pt), negligible H_2_ can be detected by the gas chromatography, which is mostly attributed to the sluggish proton coupling on the COF surface.^[^ ^25^ ^]^ In order to promote the dissociation of photogenerated excitons and proton coupling, a certain amount of Pt was in situ photodeposited on the COF surface (Pt@NKCOFs) during the activity test (Hexachloroplatinic acid was adsorbed by the COF for 2 h). When the content of deposited Pt on NKCOF‐113‐M (10 mg) was optimized to be 5 wt.%, the mass normalized H_2_ evolution rate (HER) reached a maximum of 13.1 mmol g^–1^ h^–1^ (**Figure** **5**a; Figure S10, Supporting Information). The excessive Pt loading can hinder light absorption and are always the most important site for charge‐carrier recombination.^[^ ^26^ ^]^ Also, the dosage of the photocatalyst could affect the photocatalytic performance because the higher photocatalyst mass would scatter light (Figure 5b). After optimization, the HER can reach to optimum 630 µmol h^–1^ by 50 mg NKCOF‐113‐M with 5 wt.% Pt loading. When replaced TEOA with ascorbic acid (AA), the photocatalytic activity is greatly reduced (Figure S11, Supporting Information), indicating the important role of sacrificial agents.

The apparent quantum yield (AQY) is a crucial parameter for assessing the apparent efficiency of solar to hydrogen by the photocatalysts. The light conversion efficiency of Pt@NKCOF‐113‐M under different wavelength light irradiation (420 ± 10, 450 ± 10, 475 ± 10, 500 ± 10, 550 ± 10, and 600 ± 10 nm) are shown in Figure 5c. The AQY values plotted with increasing the wavelength exhibited a volcano shape centered at 475 nm, which is roughly consistent with the UV–vis DRS of NKCOF‐113‐M. In the short wavelength region (*λ* < 475 nm), the abnormal trend of AQY values with wavelength is caused by the low penetration of short wavelength light. The AQY results proved that the light conversion efficiency is highly dependent on the light‐harvesting level for Pt@NKCOF‐113‐M. The AQY values at 420, 450, 475, 500, 550, and 600 nm were 45.2%, 52.8%, 56.2%, 21.2%, 7.8%, and 6.5%, respectively. The exceptional AQY value obtained for Pt@NKCOF‐113‐M at 475 nm is ≈70 times higher than the Pt modified polymer carbon nitride (PCN) benchmark (The AQY is ≈0.8% at 475 nm consistent with the reported value,^[^ ^25^ ^]^ Figure S12, Supporting Information). Furthermore, this value remarkably exceeded the AQY values of the most promising Pt@COFs photocatalysts reported so far (Table S1, Supporting Information), even for the hybrid COF‐based materials. The long‐term stability and reproducibility of the H_2_ evolution using the optimized Pt@NKCOF‐113‐M were analyzed by consecutive photocatalytic experiments under visible light irradiation (Figure 5d). Considering the consumption of TEOA, the recovered photocatalyst was dispersed in a freshly mixed solution of deionized water and TEOA for each photocatalytic test. The steady HER without significant decay during the continuous irradiation (12 h) for Pt@NKCOF‐113‐M, indicating its excellent stability and recoverability.

In order to further verify the effect of spatial distance regulation between Bpy and TA units, we measured the photocatalytic performances of the other two COFs under the same conditions as NKCOF‐113‐M (Figure 5e). We found the trend of the HER to be Pt@NKCOF‐113‐M > Pt@NKCOF‐112‐M > Pt@NKCOF‐114‐M. For comparison, all data of physical/chemical properties and photocatalytic HER for these COFs were collected in **Table** **1**. This result suggested that the moderate distance between Bpy and TA units in the COF is crucial for high photocatalytic activity. From the energy band structure, we can find that the three COFs meet the thermodynamic requirements of photocatalytic overall water splitting that is the ultimate goal in the pursuit of solar‐powered photocatalysis. Then, the water oxidation performances of the three Pt@NKCOFs were evaluated using 0.1 m Ce(NH_4_)_2_(NO_3_)_6_ as an electron sacrificial agent under visible‐light irradiation (Figure S13, Supporting Information). The photocatalytic evolved O_2_ was quantified by the standard curve (Figure S9b, Supporting Information). Pt@NKCOF‐113‐M and Pt@NKCOF‐114‐M show a similar O_2_ evolution activity with a maximal O_2_ evolution rate (OER) of ≈190 µmol h^–1^. However, no O_2_ can be detected using NKCOF‐112‐M during the photocatalytic test. Combined with the experimental results of photocatalytic H_2_ evolution, Pt@NKCOF‐113‐M was predicted to be able to drive overall water splitting. Overall water splitting proceeded under visible irradiation (*λ* > 420 nm) with initial rates of 5.2 and 2.4 µmol h^–1^ for HER and O_2_ evolution rate (OER) as shown in Figure 5f. The solar‐to‐hydrogen (STH) energy conversion efficiency was calculated to be 0.007% that was much lower than the CTF^33^. For the enhanced performance of photocatalytic overall water splitting, we have tried loading Co species (CoO_x_ and Co(OH)_2_) on the COF surface according to the reports.^[^ ^27^ ^]^ Nevertheless, Pt@NKCOF‐113‐M lost its activity for photocatalytic overall water splitting after Co species loading, which might be caused by the coordination between Bpy and Co^2+^ on the COF skeleton. Although the STH of Pt@NKCOF‐113‐M was very low concerning inorganic and organic semiconductors, it was still the only COF photocatalyst that can proceed with overall water splitting except for the CTF^33^.

**Table 1:** The physical/chemical properties and photocatalytic HER of the NKCOFs

Samples	*λ*_abs_ [nm]	*E*_g_ [eV]	*λ*_em_ [nm]	Stokes Shift [nm]	*τ*_avg_ [ns]	*E*_fb_ [V]	*E*_VB_ [eV]	*E*_CB_ [eV]	HER [umol h^−1^]
**NKCOF‐112‐M**	516.7	2.4	556.5	150	2.5	−0.06	−6.52	−4.12	140
**NKCOF‐113‐M**	539.2	2.3	545.8	130	2.8	−0.20	−6.36	−4.06	630
**NKCOF‐114‐M**	539.2	2.3	543.7	130	2.2	−0.03	−6.45	−4.15	58

***λ*_abs_**: UV–vis absorption edge;

***λ*_em_**: Fluorescence emission upon excitation at 400 nm;

***τ*_avg_**: Average fluorescence lifetime;

***E*_fb_**: The flat‐band potentials (pH 7, versus RHE);

***E*_VB_**: The valence band maximums were determined from the UPS (vs vacuum level);

***E*_CB_**: The conduction band minimums were calculated from EVB and optical gap (vs vacuum level);

HER: The photocatalytic H_2_ evolved rates of the Pt‐modified NKCOFs.

**Figure 6:**
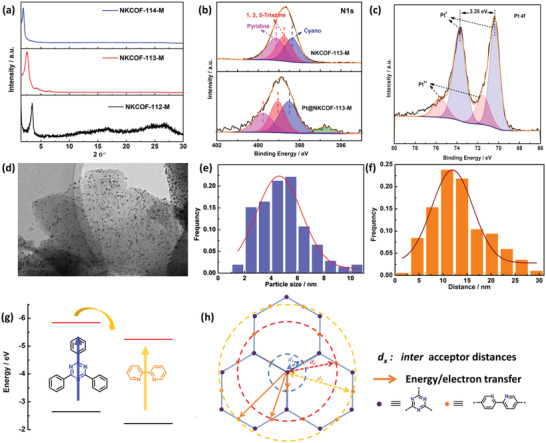
a) The PXRD patterns of the three Pt@NKCOFs after photocatalytic tests; b) the comparison of high‐resolution N 1s XPS curves of NKCOF‐113‐M and Pt@NKCOF‐113‐M; c)the high‐resolution Pt 4f XPS curves of Pt@NKCOF‐113‐M; d) The TEM of Pt@NKCOF‐113‐M; e) the related size distribution of Pt nanoparticles and f) Statistical distances between centers of neighboring Pt nanoparticles on the surface of NKCOF‐113‐M; g) the energy/electron transfer path by DFT calculation (HOMO: black line, LUMO: red line); h) schematic illustration of the relationship between *L*_D_ and Pt nanoparticles size in the COFs.

As demonstrated in previous works, Pt nanoparticles can be photodeposited on the surfaces of COFs as the co‐catalysts, which can be used as probes to track the reactive sites. The chemical element states of the COFs were characterized by X‐ray photoelectron spectroscopy (XPS). The high‐resolution N 1s and C 1s spectra for all three COFs were studied to distinguish the N and C elements with the specific chemical environment (Figure S14, Supporting Information). After Pt loading, the binding energy of N 1s electrons in Bpy and TA were shifted to high values (**Figure** **6**b), indicating that the coordination might occur between Pt and N sites on Bpy and TA units. In the high‐resolution Pt 4f spectra, Pt@NKCOF‐113‐M exhibited two pair peaks corresponding to the Pt^0^ and Pt^4+^ state (Figure 6c), which proved that Pt^4+^ is coordinated with the N sites and then reduced by the photoelectrons. By DFT calculation, we can infer that Pt^0^ is preferentially coordinated with the two N sites on the Bpy unit during the photocatalytic tests according to their adsorption energy (Figure S15, Supporting Information), which is consistent well with the XPS results. The above experimental results show the *π*‐electron delocalization at the N sites for the reduction of Pt^4+^ and the generation of H_2_.

The size and distribution of Pt nanoparticles on the COFs surface reflected the distribution of photocatalytic reaction sites. The smaller size and the denser distribution suggest the more effective reaction sites. The TEM images (Figure 6d—f; Figure S16, Supporting Information) show the highly dispersed Pt nanoparticles on the COFs surfaces with size distributions centered at 28.63 ± 0.99, 4.61 ± 0.17, and 16.89 ± 0.71 nm for NKCOF‐112‐M, NKCOF‐113‐M, and NKCOF‐114‐M, respectively. The distances between the neighboring Pt nanoparticles were 59.89 ± 2.21, 11.94 ± 0.47, and 30.06 ± 0.78 nm for NKCOF‐112‐M, NKCOF‐113‐M, and NKCOF‐114‐M. Lattice fringes were observed for Pt nanoparticles with the *d*‐spacing value of 0.23 nm, indicating the presence of Pt (111) planes (Figure S17, Supporting Information). Notably, the 1D channels (see the white lines as the guide in Figure S18a, Supporting Information) and the hexagonal pores (Figure S18b, Supporting Information) further confirmed the maintained high crystallinity of the COFs during the photocatalytic tests. Therefore, the size and distribution of Pt nanoparticles on NKCOF‐113‐M present a key role in the highest photocatalytic activity.

The photogenerated energy/electron transfer paths were further investigated by DFT calculations. Bpy and TA as the acceptor units were installed in the highly crystalline framework, which ensures the energy/electron transfer between two units. The distance between the two acceptors was tuned by the phenyl units, which led to the formation of the three TA model molecules (M1–M3) and one Bpy model molecule (M4) (Figure S19, Supporting Information). DFT theoretical simulations of the four acceptors showed different electron densities on the HOMO and LUMO levels (Figure S19a, Supporting Information). The DFT calculated results indicated that the energy/electron transfer from TA to Bpy in the three COFs owing to their energy levels (Figure 6g; Figure S19b,c, Supporting Information). The photogenerated excitons and electrons can easily migrate along the *π*‐conjugated plane and the *π*–*π* stacking direction.^[^ ^10^ ^]^ The coordinated Pt^4+^ at N sites was in situ reduced by the photogenerated electrons to form the Pt^0^/COF interface. Due to the dielectric constant, the photogenerated exciton dissociates at Pt^0^/COF interface, and the photoexcited *π*‐electrons flow to Pt° for H_2_ generation. The anchoring sites (N sites, Figure S14, Supporting Information) of Pt^0^ are the key to the size and distribution of Pt nanoparticles.

According to the above TEM results, the Pt nanoparticles on the three COFs surfaces varied greatly, which might also be related to the intrinsic diffusion distance (*L*_D_) of photogenerated carriers. The intrinsic carrier transport characteristics of the COFs were measured by the Hall effect at 300 K,^[^ ^30^ ^]^ as shown in Table S7 (Supporting Information). The n‐type semiconductive natures of the three COFs were further evidenced by the negative Hall coefficient, consisting of the Mott–Schottky measurements. Thus, the *L*_D_ of the COFs was obtained by the equation of *L*_D_ *=* *(K*_B_ *T/e* *×* *µ* × *τ*)^1/2^ (where *K*_B_ is Boltzmann's constant, *e* the electron charge, *µ* the Hall mobility, *τ* the carrier lifetime, and *T* the sample temperature),^[^ ^31^ ^]^ as shown in **Table** **2**. The *L*_D_ of the three COFs was almost equal. Therefore, the smaller space between Pt nanoparticles on NKCOF‐113‐M is conducive to the rapid migration of the excitons to the Pt/COF interface and dissociation, so as not to de‐excitation during the transfer process. Interestingly, *L*_D_ correlated to the size of Pt nanoparticles. The distances between Bpy and TA in COFs were divided into *d*_1_, *d*_2_ *_,_* and *d*_3_ (Figure 6h and Table 1). *L*_D_ of NKCOF‐112‐M is almost equal to the longest distance (*d*_3_) between Bpy and TA, suggesting that the photogenerated electrons may randomly overflow from all acceptor units to cause the unlimited growth of Pt nanoparticles (the largest particle size). *L*_D_ is matched with *d*_2_ in NKCOF‐113‐M, indicating the Pt nanoparticles were confined growth in an area with a radius of ≈3.4 nm. Sufficient adsorption sites and growth confinement space resulted in the monodisperse Pt nanoparticles with a size of 4.61 nm on the NKCOF‐113‐M surface. As for NKCOF‐114‐M, *L*_D_ only satisfies the shortest distance (*d*_1_). Contrary to the expected smallest Pt nanoparticles, the experimental Pt nanoparticle's size was much larger. This must be due to that insufficient adsorption sites in the confined space leading to the aggregation of the Pt nanoparticles. Therefore, the proper spatial distance between Bpy and TA units in NKCOF‐113‐M contributes to the confined growth of Pt nanoparticles. During the photocatalytic reaction, the target reaction and carrier recombination are in dynamic equilibrium. Small size and high‐density Pt nanoparticles (Note: The amount of Pt on the surface of the photocatalyst is not enough to block the light) ensure sufficient active sites for H_2_ evolution, which accelerate the consumption of photogenerated electrons for target reaction, so as to avoid the recombination between photogenerated electrons and holes. In other words, both the small size of Pt nanoparticles and the shorter spacing between Pt nanoparticles were controlled by the inter Bpy and TA distance in NKCOF‐113‐M to promote the dissociation of the excitons and prevent the carriers recombination.

**Table 2:** The inter acceptors distances and the charge carrier diffusion lengths

Sample	Inter acceptors’ Distance [nm]			Diffusion lengths [nm]
	*d*_1_	*d*_2_	*d*_3_	*L*_D_
**NKCOF‐112‐M**	0.87	2.26	3.18	1.8–2.9
**NKCOF‐113‐M**	1.31	3.41	4.81	1.7–3.2
**NKCOF‐114‐M**	1.75	4.71	6.2	1.5–2.9

## 3 Conclusion

In conclusion, for the first time, we systematically investigated the influence of the spatial distance between the acceptors in COF skeletons on their H_2_ evolution performance. Thus, three highly crystalline, porous and robust olefin‐linked 2D COFs installed with 1,3,5‐Triazine and 2,2“‐bipyridine as electron acceptor units were fabricated using a novel melt polymerization method. We found that regulation of the spatial distance between two acceptors can tune the light‐harvesting characteristics, band structure, and excited state lifetime of the COFs. Consequently, NKCOF‐113‐M with a moderate distance between Bpy and TA units exhibited photocatalytic H_2_ evolution activity with AQY as high as 56.2% at 475 nm, the second high value among all COF photocatalyst. Interestingly, NKCOF‐113‐M also achieved the photocatalytic overall water splitting under visible light, which was rarely reported by polymer semiconductors in photocatalytic H_2_ evolution. Both the diverse optical and electrical experiments, as well as DFT calculation, revealed that the outstanding performance of NKCOF‐113‐M was attributed to the intrinsically longer excited‐state lifetime and higher photocurrent response in comparison with NKCOF‐112‐M and NKCOF‐114‐M. Moreover, the TEM images revealed that the confined growth of photodeposited Pt nanoparticles within charge carrier diffusion length (*L*_D_) can dramatically improve the photocatalytic H_2_ evolution. This work not only demonstrates a strategy of inter acceptors” spatial distance regulation in 2D COFs to perform photocatalytic H_2_ evolution, but also guild the future study of organic semiconductor‐based photocatalysis.

## Conflict of Interest

The authors declare no conflict of interest.

## Supporting information

Supporting Information

## Data Availability

The data that support the findings of this study are available in the supplementary material of this article.
