# Supplementary material for: Spatial Regulation of Acceptor Units in Olefin‐Linked COFs toward Highly Efficient Photocatalytic H2 Evolution
Source: Adv Sci (Weinh). 2022 Aug 18;9(29):2203832. doi: 10.1002/advs.202203832 (PMC9561862; doi:10.1002/advs.202203832)
Supplement: Supplementary file 1 — Supporting Information [file ADVS-9-2203832-s001.pdf]

ADVANCED SCIENCE
Open Access

Supporting Information

for *Adv. Sci.*, DOI 10.1002/advs.202203832

Spatial Regulation of Acceptor Units in Olefin-Linked COFs toward Highly Efficient Photocatalytic H_2 Evolution

Zhengfeng Zhao, Xuepeng Chen, BaoYing Li, Shu Zhao, Liwei Niu*, Zhenjie Zhang and Yao Chen**

Supporting Information

Spatial Regulation of Acceptor Units in Olefin-linked COFs towards Highly Efficient Photocatalytic H_2 Evolution

Zhengfeng Zhao, Xuepeng Chen, BaoYing Li, Shu Zhao, Liwei Niu*, Zhenjie Zhang, Yao Chen**

Zhengfeng Zhao, BaoYing Li, Liwei Niu

School of Chemistry and Chemical Engineering, Qilu University of Technology (Shandong Academy of Sciences), Jinan 250353, China

Xuepeng Chen, Prof. Z. Zhang, Prof. Y. Chen

State Key Laboratory of Medicinal Chemical Biology, College of Pharmacy, Nankai University, Tianjin 300071, China

Shu Zhao

Institute of Advanced Battery Materials and Devices, Faculty of Materials and Manufacturing, Beijing University of Technology, Beijing, 100124, P. R. China

*Corresponding author.

Email: mse_zhengfengzhao@163.com; niuliwei@qlu.edu.cn; chenyao@nankai.edu.cn

Section1. Experimental details.

General. All materials were commercially available and used without further purification. All solvents were of analytical grade and used without further purification. ***2***,4,6-trimethyl-1,3,5-triazine (TA-CH_3), [2,2'-bipyridine]-5,5'-dicarbaldehyde (Bpy-CHO) and benzoic anhydride were purchased from Energy Chemical (97%), HEOWNS (98%) and Aladdin (98%), respectively.

Characterization. ^{1}H and ^{13}C nuclear magnetic resonance (NMR) was recorded on Bruker 400 M NMR spectrometer. Magic angle spinning (MAS) solid-state Nuclear magnetic resonance (ssNMR) spectra were recorded using a Bruker Avance III WB 400 spectrometer. PXRD patterns of all the materials were collected at ambient temperature on Rigaku Dmax 2500 diffractometer using Cu Kα (λ=1.5418 Å) radiation, with a scan speed of 5 sec/step, a step size of 0.01° in 2θ, and a 2θ range of 1.5° to 30°. Fourier transform infrared spectra (FT-IR) were recorded on Nicolet IS10. X-ray photoelectron spectroscopy (XPS) measurements were performed on an X-ray photoelectron spectrometer (Thermo Scientific ESCALAB Xi+). Low pressure gas adsorption measurements were measured with BELSORP MaxII for surface area and pore size analysis. A liquid nitrogen bath was used for the N_2 measurements at 77 K and CO_2 sorption isotherms were measured at 298 K. UV-vis diffused reflectance spectra were measured on a UV spectrophotometer (UV-2700, Shimadzu). Steady-state photoluminescence (PL) and excited-state decay spectra were measured on FLS1000-Edinburgh Instruments. Photoelectrochemical characterization was carried out on a CHI600E workstation. Hydrophobic angle of the COFs were tested on a SINDIN SDC-200. The transmission electron microscopy (TEM, JEOL-JEM 2100 F) was used to analyze the morphologies of NKCOFs. Hall effect was performed by Ecopia HMS-7000 at 300 K.

Photocatalytic H_2 evolution

A flask was charged with a given mass of the COFs powder, 10 mL Triethanolamine (TEOA), 90 mL water solution, and a certain proportion hexachloroplatinic acid (50mM aqueous solution) as a Pt precursor. Then the resulting suspension was transferred into a Pyrex top-irradiation reaction vessel connected to a closed gas system. The resulting suspension was ultrasonicated for 20 min before degassing. The reaction mixture was evacuated several times to ensure complete removal of air and then was illuminated with a 300 W Xe light source (λ > 420 nm) with appropriate filters for the period specified. The temperature of the reaction solution was maintained at room temperature by the flow of cooling water. The evolved gases were analyzed by gas chromatography with argon as the carrier gas. Hydrogen was detected with a thermal conductivity detector (TCD) referencing standard gas with a known concentration of hydrogen. After the photocatalysis experiment, the COFs were recovered by washing with methanol before drying at 120 °C.

Photocatalytic oxygen evolution

50 mg of Pt@COFs was well dispersed by ultrasonication in an aqueous solution (100 mL) containing, $Ce(NH_4)_2(NO_3)_6$ (0.1 mol L^{-1}) as electron acceptor and La_2O_3 (0.2 g) as pH buffer. The suspension was poured into a Pyrex top-irradiation reaction vessel connected to a closed gas system. Then it was evacuated several times to completely remove air prior to irradiation under a 300 W Xe lamp (λ > 420 nm). The temperature of the reaction solution was maintained at room temperature by the flow of cooling water. The evolved gases were analyzed by gas chromatography with argon as the carrier gas. Oxygen was detected with a thermal conductivity detector (TCD) referencing against standard gas with a known concentration of oxygen.

Photocatalytic overall water splitting

50 mg of Pt@COFs was well dispersed by ultrasonication in 100 mL of deionized water.

The suspension was poured into the Pyrex top-irradiation reaction vessel connected to a closed gas system. Then it was evacuated several times to completely remove air prior to irradiation under a 300 W Xe lamp (λ > 420 nm). The temperature of the reaction solution was maintained at room temperature by the flow of cooling water. The evolved gases were analyzed by gas chromatography with argon as the carrier gas.

The apparent quantum yield (AQY) measurements

The apparent quantum yield (AQY) for H_2 evolution was measured usia ng 300 W Xe lamp with band pass filter of 420 ± 10 nm, 450 ± 10 nm, 475 ± 10 nm, 500 ± 10 nm, 550 ± ,10 nm and 600 ± 10 nm the intensities were 4.8, 6.2, 6.4, 5.4, 5.7 and 4.8 mW cm^{-2}, respectively. The irradiation area was controlled as 3.14×2.6^2 cm^2. The AQY was calculated according to the follow Eq.: $\eta_{AQY} = \frac{N_e}{N_p} * 100\% = \frac{2*M*N_A*h*c}{S*P*t*\lambda} * 100\%$;

Where, N_e is the amount of generated electrons for H_2, N_p is the amount of incident photons, M is the amount of H_2 molecules (mol) during 0.5 hour, N_A is Avogadro constant (6.022×10^{23} mol^{-1}), h is the Planck constant (6.626×10^{-34} J·s), c is the speed of light (3×10^8 m/s), S is the irradiation area (m^2), P is the intensity of irradiation light (W /m^2), t is the photoreaction time (t=1800 s), λ is the wavelength of the monochromatic light (m).

The Solar-to-hydrogen (STH) measurements

The STH energy conversion efficiency (η) was calculated according to the following Eq.:

$$\eta_{STH} = R_H * \frac{\Delta G^0}{P*S} * 100\%;$$

where R_H, ΔG^0, P, and S denote the rate of H_2 evolution (mol s^{-1}) in photocatalytic water splitting, standard Gibbs energy of water (237.13×10^3 J mol^{-1}), intensity of simulated sunlight (0.1 W cm^{-2}), and irradiation area (21.2 cm^2), respectively.

Structure simulations. Structural modeling of all the COFs was generated using the Accelrys Materials Studio software package. The lattice model was geometry optimized using the Forcite module. Pawley refinement was applied to define the lattice parameters.

Computation methods

The calculations were carried out using the Vienna Ab Initio Simulation Package (VASP)[1] with the frozen-core projector-augmented wave (PAW) method[2]. The generalized gradient approximation in the Perdew-Burke-Ernzerhof (GGA-PBE)[3] function was employed for the exchange-correlation energy. a cutoff energy of 400 eV was selected for the plane-wave expansion. The convergence criteria for the force and electronic self-consistent iteration were set to 0.03 eV/Å and 10^{-4} eV, respectively. The Gamma k-point was used to sample the Brillion zone.

Adsorption energies were calculated according to *E*ads = *E*x/slab – [*E*slab + *E*x], where *E*x/slab is the total energy of the slab with adsorbents in its equilibrium geometry, *E*slab is the total energy of the bare slab, and *E*x is the total energy of the free adsorbents in the gas phase. Therefore, the more negative the Eads, the stronger the adsorption.

COFs Activation:

The as-synthesized olefin-linked COFs were Soxhlet extracted with tetrahydrofuran and methanol for 48 h. And then these materials were activated by supercritical CO_2 for 3 h.

Electrochemical measurements

Cyclic voltammetry measurements were performed in a typical three electrode cell system with a scan rate of 0.05 V/s. The cleaned ITO glass was coated with NKCOFs suspension solutions (1 mg/mL in EtOH and H_2O (V : V=1 : 1) with a few droplets of 5 wt% Nafion) and then dried in air as the working electrode. The Pt flake and Ag/AgCl electrodes were used as counter electrode and reference electrode, respectively. For photocurrent intensity response and flat band potential (E_{fb}) measurements, the electrolyte was changed to 0.1 M Na_2SO_4

aqueous solution, the reference electrode was altered to Ag/AgCl electrode and the light source was provided by a 300 W Xe-lamp with an ultraviolet cut-off filter (λ > 420 nm).

Synthesis of 2,4,6-Tris(4-formylphenyl)-1,3,5-triazine (TA-1Ph-CHO)

2,4,6-Tris(4-formylphenyl)-1,3,5-triazine was synthesized according to the reported literature. 2,4,6-Tris(4-bromophenyl)-1,3,5-triazine (1.48 g, 2.71 mmol) dissolved in dry THF (200 mL) under a Ar atmosphere. To the stirred solution, n-BuLi was added dropwise (2.5 M in n-hexane, 11 mL, 27.5 mmol) at −78 °C. The temperature was allowed to rise to −60 °C and stirred for 3 h. The obtained solution was treated with anhydrous N, N-dimethylformamide (DMF) (4.19 mL, 54.2 mmol) at −78 °C. The mixture was stirred overnight, while the temperature was allowed to rise to 25 °C. The mixture was acidified with aqueous 3 M HCl (46 mL). The organic volatiles were partially removed by evaporation under reduced pressure, and the product was extracted with CH_2Cl_2 and further purified by silica gel column using CH_2Cl_2 as eluent to afford colorless crystals (600 mg, 55% yield). ^1H NMR (400 MHz, Chloroform-d) δ = 10.20 (s, 3H), 8.96 (d, 6H), 8.13 (d, 6H).

Synthesis of 4',4''',4'''''-(1,3,5-triazine-2,4,6-triyl)tris(([1,1'-biphenyl]-4-carbal dehyde)) (TA-2Ph-CHO)

1,3,5-tribromobenzene (2.73 g, 5.0 mmol), 4-formylphenylboronic acid (2.40 g, 16.0 mmol), $Pd(PPh_3)_4$ (0.58 g, 0.5 mmol) and K_2CO_3 (5.53 g, 40 mmol) dissolved in mixed solvent (water : 1,4-dioxane = 1:4, v:v) (200 mL) under a Ar atmosphere. The resulting suspension was heated at 100 ℃ and reflux for three days. After being cooled to room temperature, a large amount of water was poured into the solution. The precipitate was filtered and washed with acetone, water and methanol. The resulting solid was recrystallized in 1,4-dioxane, the desired product as off-white solid. (1.5 g, 47 %). 1H NMR (400 MHz, Chloroform-*d*) δ = 10.04 (s, 3H), 8.86 (d, 6H), 7.97 (d, 6H), 7.82 (d,12H).

Synthesis of 5,5′-bis(cyanomethyl)-2,2′-bipyridine (BPy-CN)

5,5′-bis(cyanomethyl)-2,2′-bipyridine (BCBPy) was synthesized according to the reported literature.[4] A 100 mL round-bottom flask was charged with $NiCl_2·6H_2O$ (0.12 g, 0.5 mmol) and DMF (20 mL). The resulting solution was stirred and heated to 40 °C, and then 2-(6-chloropyridin-3-yl) acetonitrile (10 mmol), anhydrous LiCl (0.43 g, 10 mmol), and zinc dust (0.78 g, 12 mmol) were added. When the temperature rose to 50 °C, a grain of iodine crystal and two drops of acetic acid were added to the mixture. The mixture was stirred at 55-60 °C for 2-3 h until complete conversion of 2-(6-chloropyridin-3-yl) acetonitrile. The cooled reaction mixture was added to 1 N HCl aqueous solution (15 mL) to consume the remaining zinc dust. The resulting mixture was made alkaline with aqueous ammonia (25%) and taken up with CH_2Cl_2. The organic layers were collected, dried over anhydrous Na_2CO_3, and concentrated. The crude material was purified by flash chromatography to give the desired colorless crystals (500 mg, 84% yield). ^{1}H NMR (400 MHz, Chloroform-*d*) δ = 8.65 (d, 1H), 8.46 (d, 1H), 7.86 (dd, 2.4, 1H), 3.85 (s, 2H).

Synthesis of NKCOF-112-M.

A 10 mL pyrex tube was charged with ***2***,4,6-trimethyl-1,3,5-triazine (TA-CH_3) (12.3 mg, 0.1 mmol), [2,2'-bipyridine]-5,5'-dicarbaldehyde (Bpy-CHO) (31.8 mg, 0.15 mmol) and benzoic anhydride (45 mg, 0.2 mmol). This mixture was degassed through three freeze-pump-thaw cycles, sealed under vacuum. Then the tube was transferred into an oven to heat at 200 °C for 3 days yielding a yellow solid that was washed with tetrahydrofuran and methanol in a Soxhlet extractor for 48 h (yield ~90%). **NKCOF-112-M** was then activated by drying under vacuum at 80 ˚C for 12 h.

Synthesis of NKCOF-113-M.

A 10 mL pyrex tube was charged with 2,4,6-Tris(4-formylphenyl)-1,3,5-triazine (TA-1Ph-CHO) (12.6 mg, 0.032 mmol), 5,5'-bis(cyanomethyl)-2,2'-bipyridine (BPy-CN) (11.3 mg, 0.048 mmol) and benzoic anhydride (22.5 mg, 0.1 mmol). This mixture was degassed through three freeze-pump-thaw cycles, sealed under vacuum. Then the tube was transferred into an oven to heat at 200 °C for 3 days yielding a yellow solid that was washed with tetrahydrofuran and methanol in a Soxhlet extractor for 48 h (yield ~95%). **NKCOF-113-M** was then activated by drying under vacuum at 80 ˚C for 12 h.

Synthesis of NKCOF-114-M.

A 10 mL pyrex tube was charged with 4',4''',4'''''-(1,3,5-triazine-2,4,6-triyl)tris(([1,1'-biphenyl]-4-carbaldehyde)) (TA-2Ph-CHO) (19.9 mg, 0.032 mmol), 5,5'-bis(cyanomethyl)-2,2'-bipyridine (BPy-CN) (11.3 mg, 0.048 mmol) and benzoic anhydride (22.5 mg, 0.1 mmol).This mixture was degassed through three freeze-pump-thaw cycles, sealed under vacuum. Then the tube was transferred into an oven to heat at 200 °C for 3 days yielding a yellow solid that was washed with tetrahydrofuran and methanol in a Soxhlet extractor for 48 h (yield ~95%). **NKCOF-114-M** was then activated by drying under vacuum at 80 ˚C for 12 h.

Synthesis of polymeric carbon nitride (PCN). Urea powder of 10.0 g was put into an alumina crucible with a cover and then heated to 550 °C in a muffle furnace for 3 h. PCN was obtained after cooling down to room temperature.

Section 2. Supporting Figures and tables.

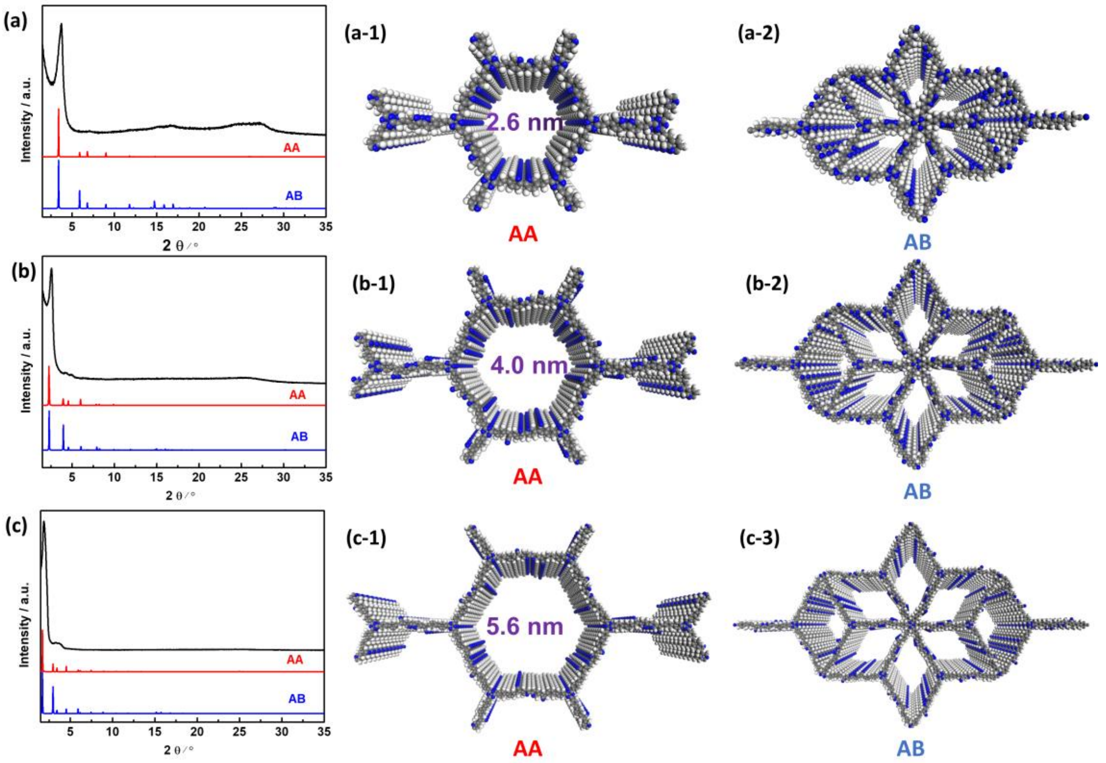

Figure S1. PXRD patterns of the olefin-linked COFs: experimentally observed (Black), simulated using eclipsed AA-stacking (Red) and staggered AB stacking (Blue) models: (a) NKCOF-112-M, (b) NKCOF-113-M, (c) NKCOF-114-M; The eclipsed AA stacking models of (a-1) NKCOF-112-M, (b-1) NKCOF-113-M, (c-1) NKCOF-114-M; The staggered AB stacking models of (a-2) NKCOF-112-M, (b-2) NKCOF-113-M, (c-2) NKCOF-114-M.

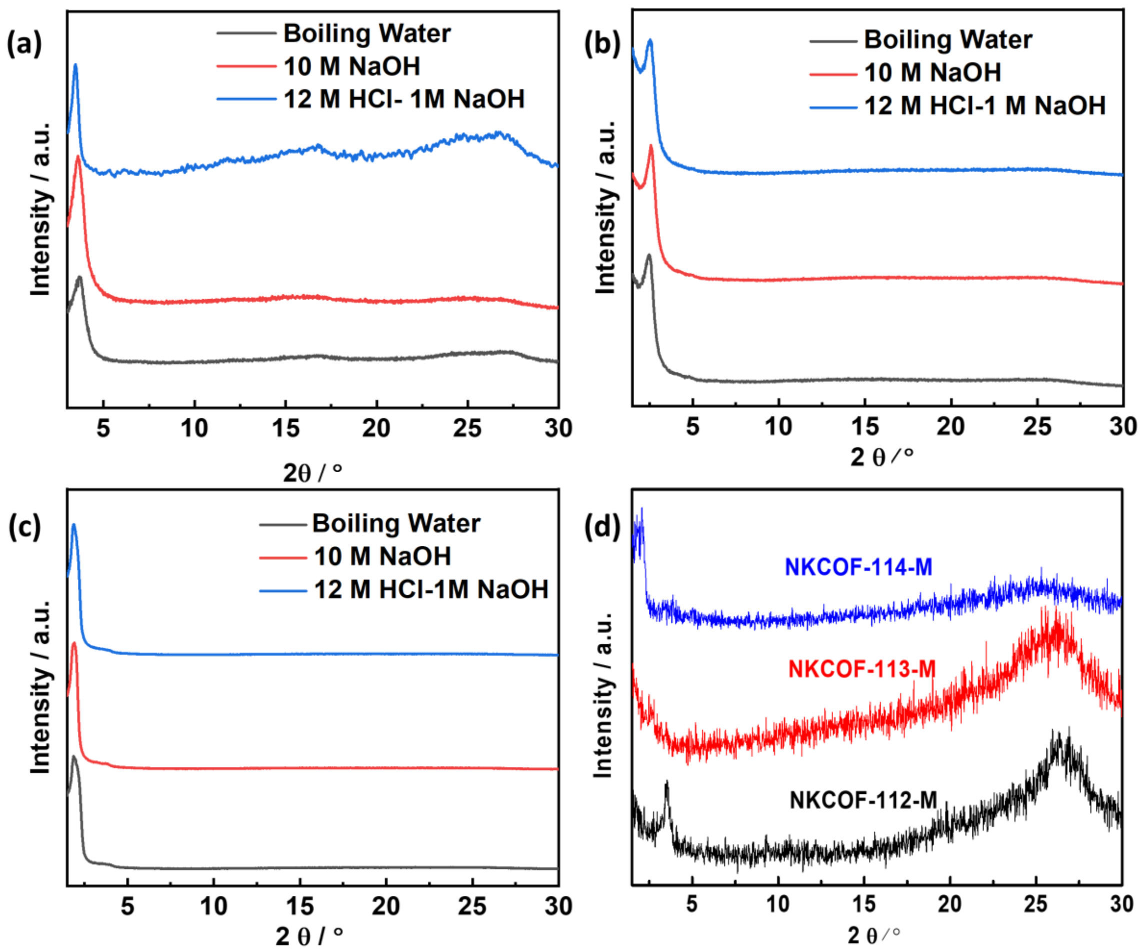

Figure S2. PXRD patterns of the olefin-linked COFs after various treatments: (a) NKCOF-112-M, (b) NKCOF-113-M, (c) NKCOF-114-M; (d) PXRD pattern of the olefin-linked COFs treated by 12 M HCl aqueous solution. The crystallinity of the COFs was influenced by concentrated HCl due to the protonation of the framework. 12M HCl-1M NaOH means after treating with 12 M HCl, 1M NaOH was used to treat the sample. The results showed the crystallinity for all COFs could be recovered due to the deprotonation.

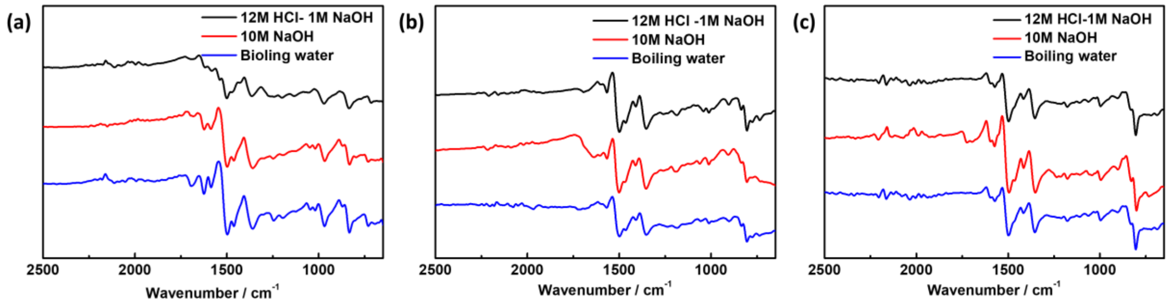

Figure S3. The FT-IR spectra of the olefin linked COFs after various treatments: (a) NKCOF-112-M; (b) NKCOF-113-M; (c) NKCOF-114-M.

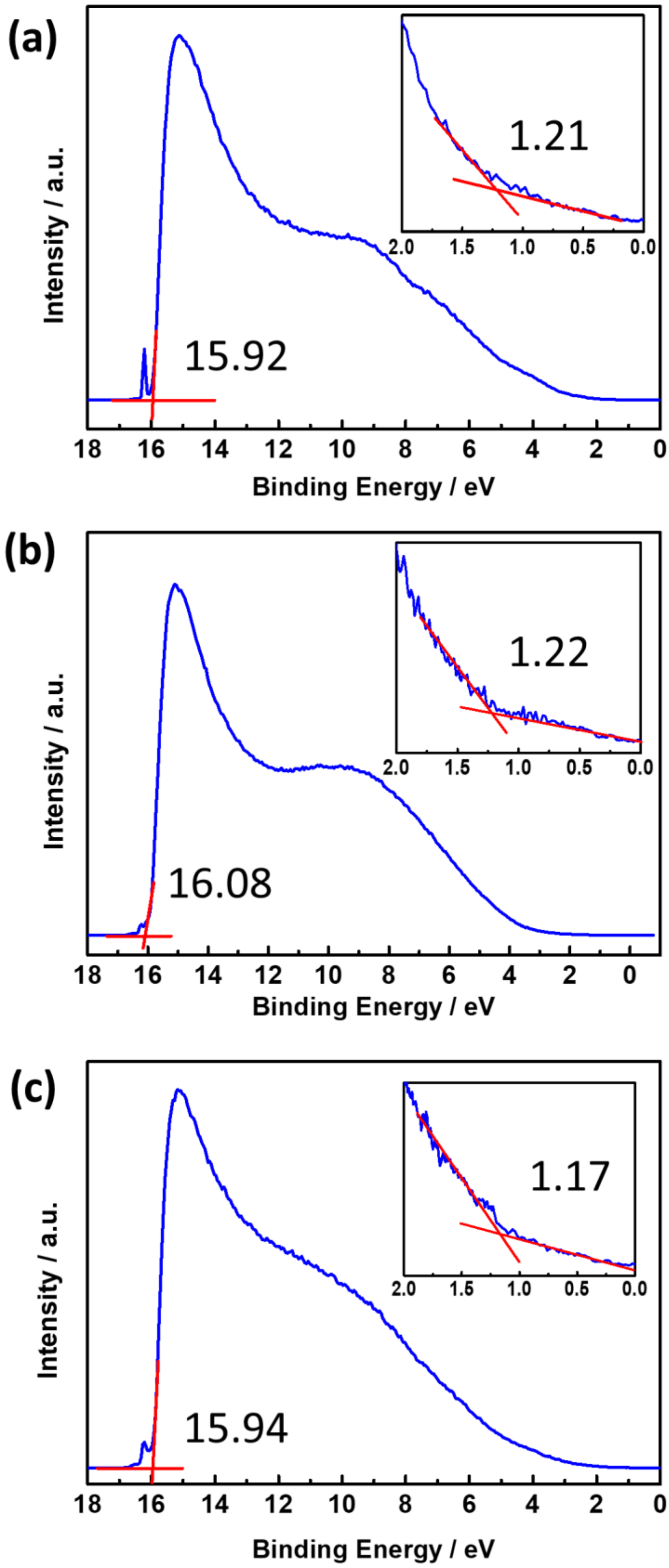

Figure S4. Ultraviolet photo-electron spectroscopy (UPS) of (a) NKCOF-112-M, (b) NKCOF-113-M, (c) NKCOF-114-M.

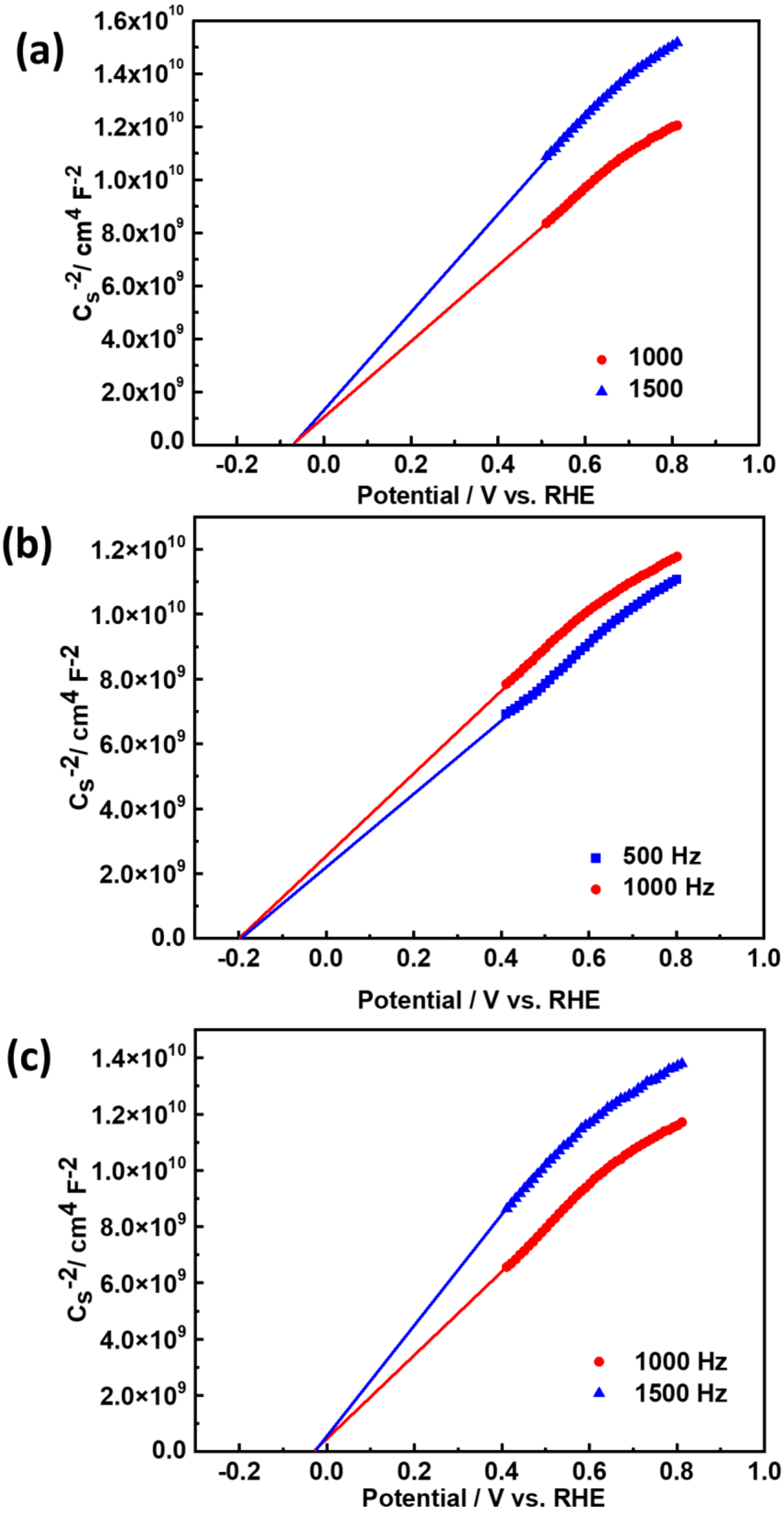

Figure S5. The Mott-Schottky plots for (a) NKCOF-112-M, (b) NKCOF-113-M and (c) NKCOF-114-M at different frequencies.

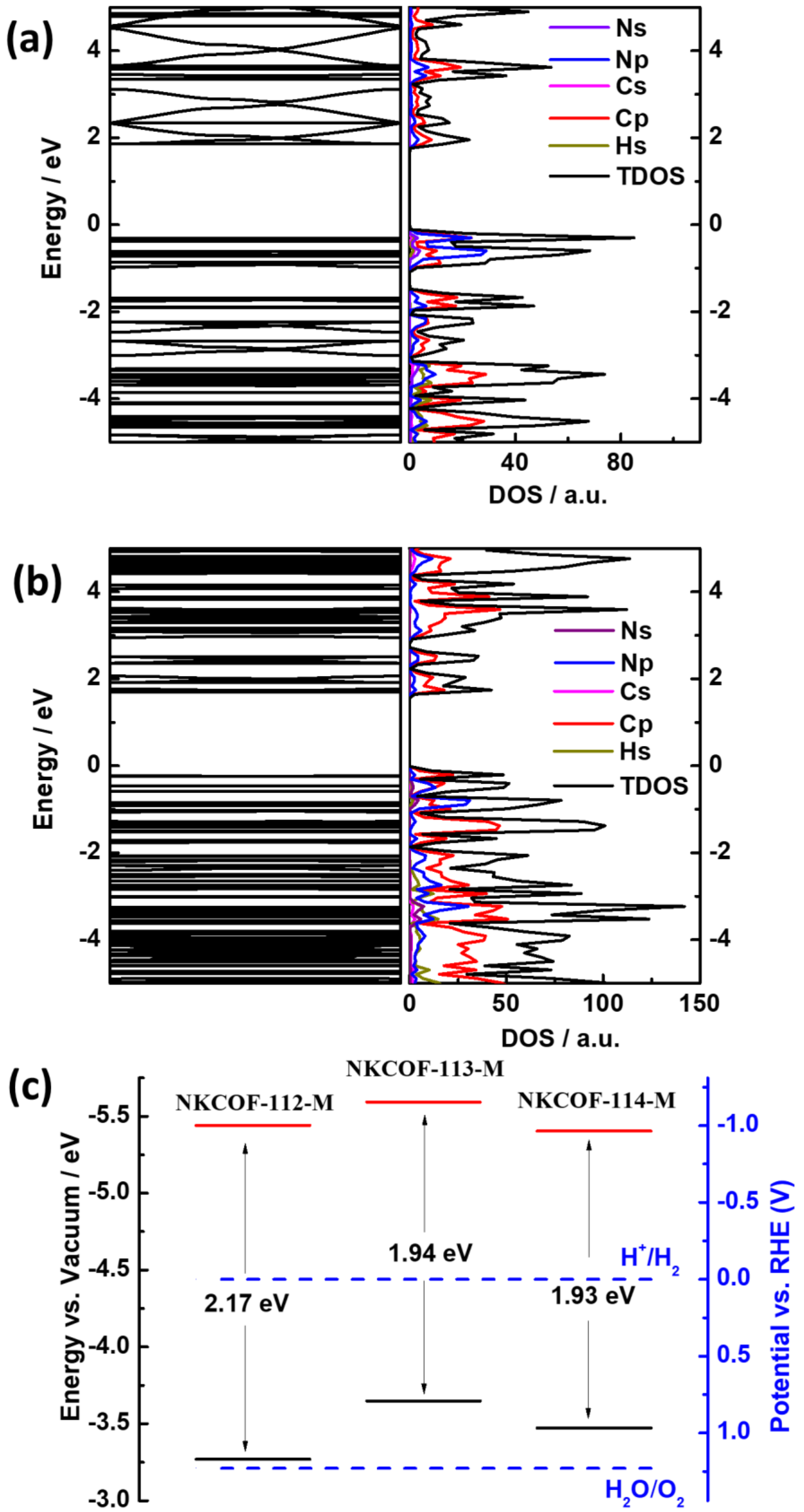

Figure S6. Electronic properties of (a) NKCOF-112-M and (b) NKCOF-114-M using DFT calculations and corresponding density of states (DOS); (c) The calculated energy levels of the COFs.

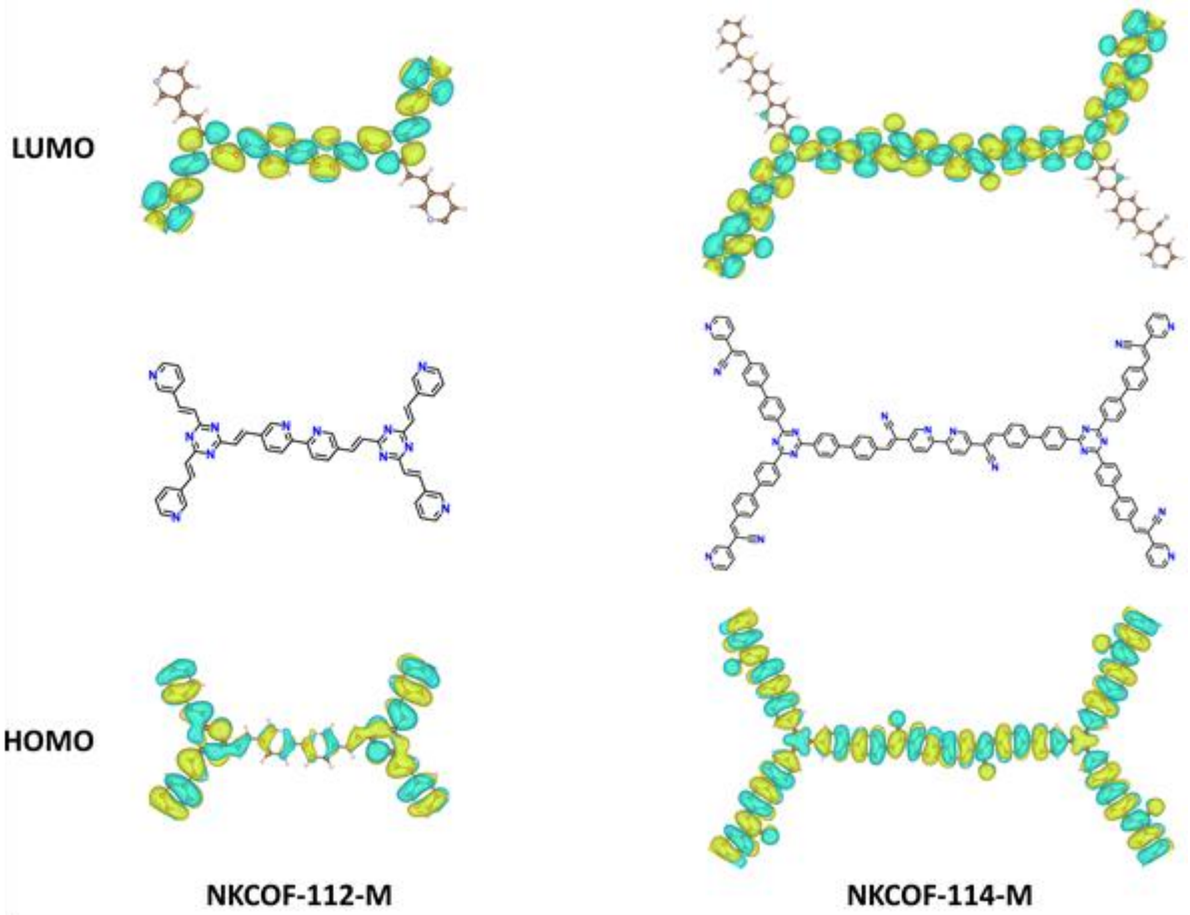

Figure S7. DFT optimized molecular orbital plots **(**HOMO and LUMO) of NKCOF-112-M and NKCOF-114-M.

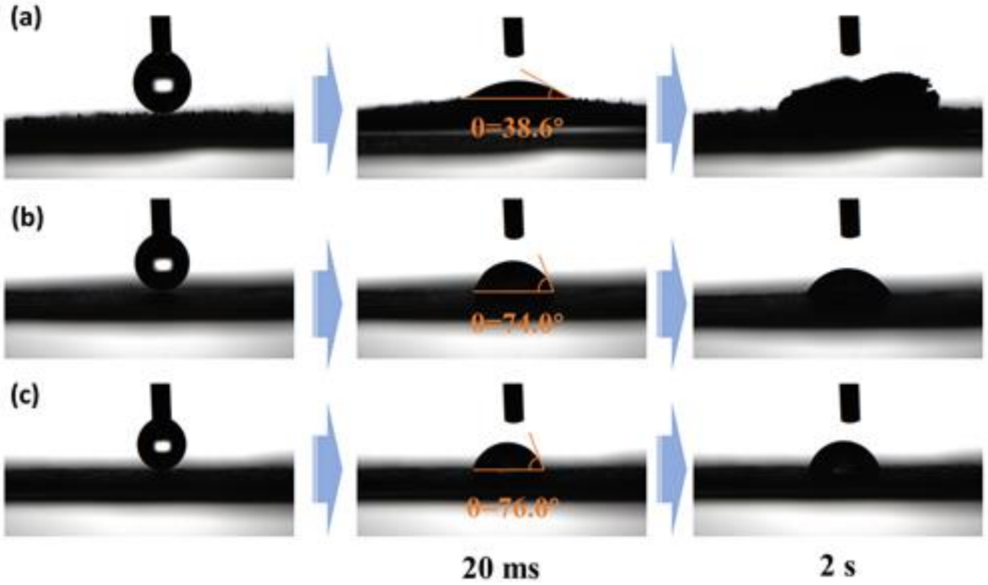

Figure S8. Hydrophobic angle measurement of (a) NKCOF-112-M; (b) NKCOF-113-M; (c) NKCOF-114-M. (NKCOF-112-M absorbed water drops in 2 s.)

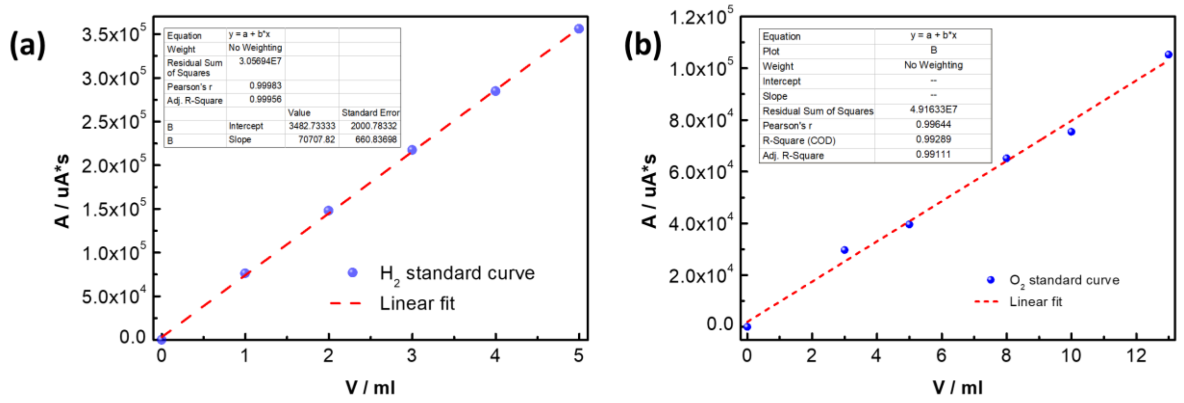

Figure S9. The standard curves of (a) H_2 and (b) O_2.

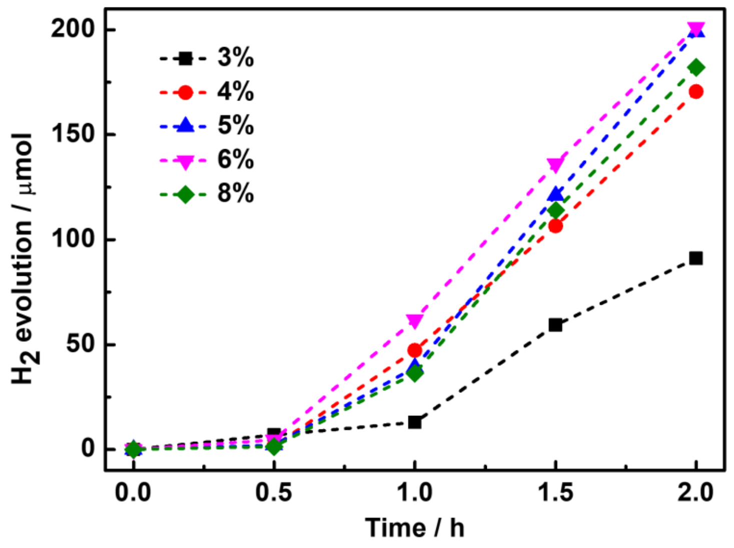

Figure S10. Time course H_2 evolution under visible light ($\lambda > 420$ nm) irradiation using 10 mg of NKCOF-113-M with different Pt loading (wt%).

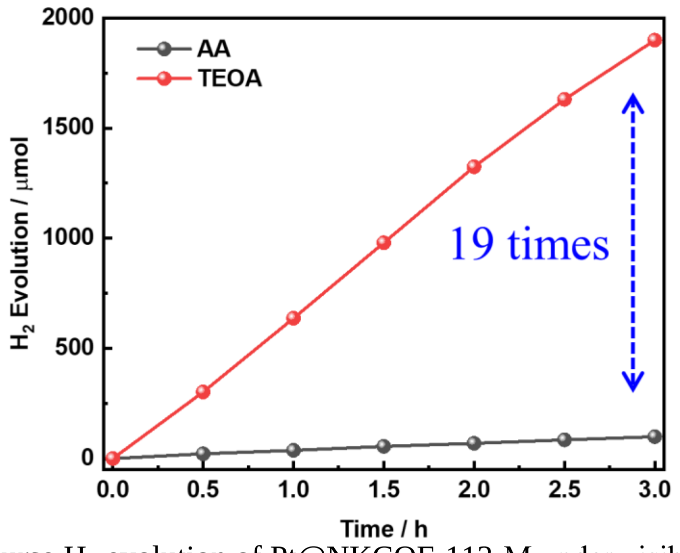

Figure S11. Time course H_2 evolution of Pt@NKCOF-113-M under visible light ($\lambda > 420$ nm) irradiation using different sacrificial agents (AA: ascorbic acid; TEOA: Triethanolamine). Conditions: 50 mg of NKCOF-113-M with 5 wt% Pt loading at room temperature.

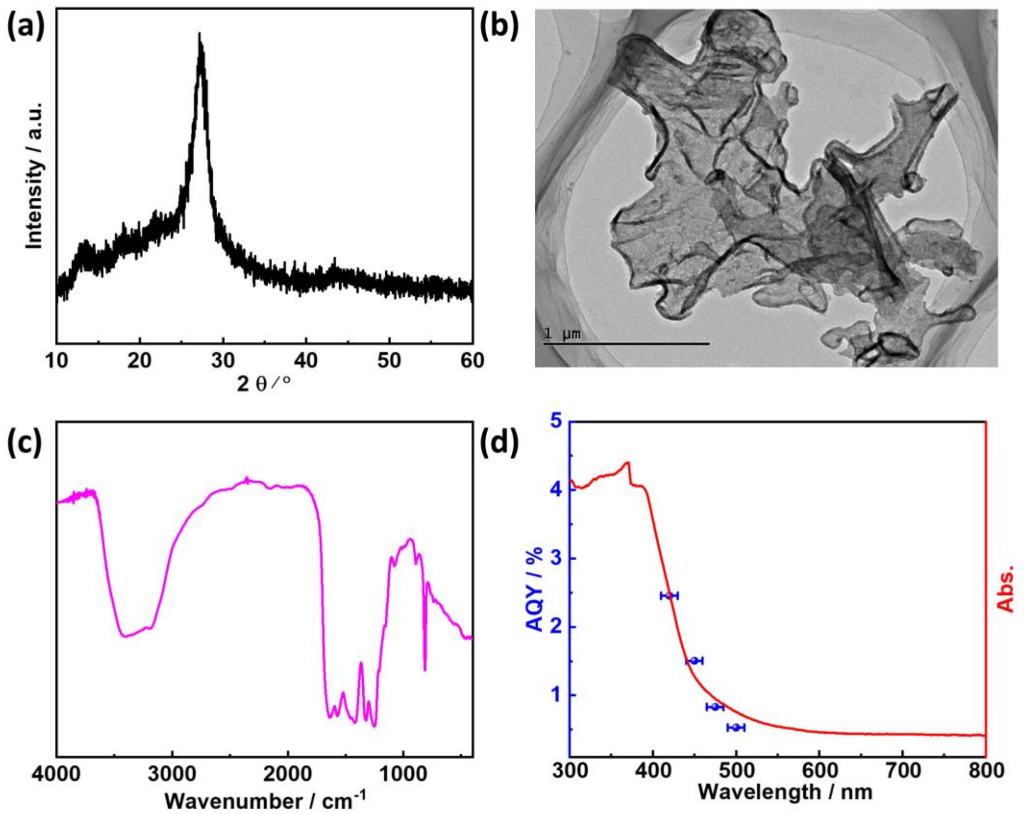

Figure S12. (a) PXRD, (b)TEM, (c) FT-IR and (d) AQY values of PCN. (The conditions of photocatalytic H_2 evolution are same as the NKCOF-113-M.)

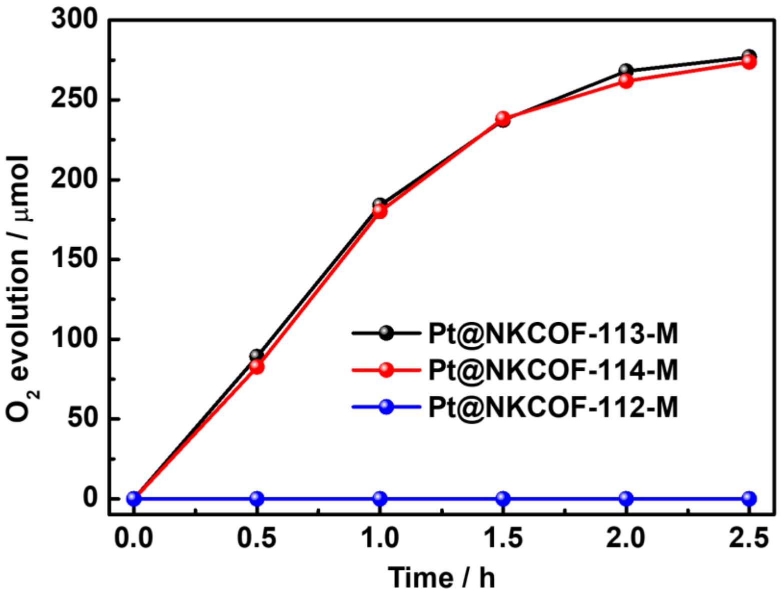

Figure S13. Time course O_2 evolution under visible light ($\lambda > 420$ nm) irradiation using 50 mg samples.

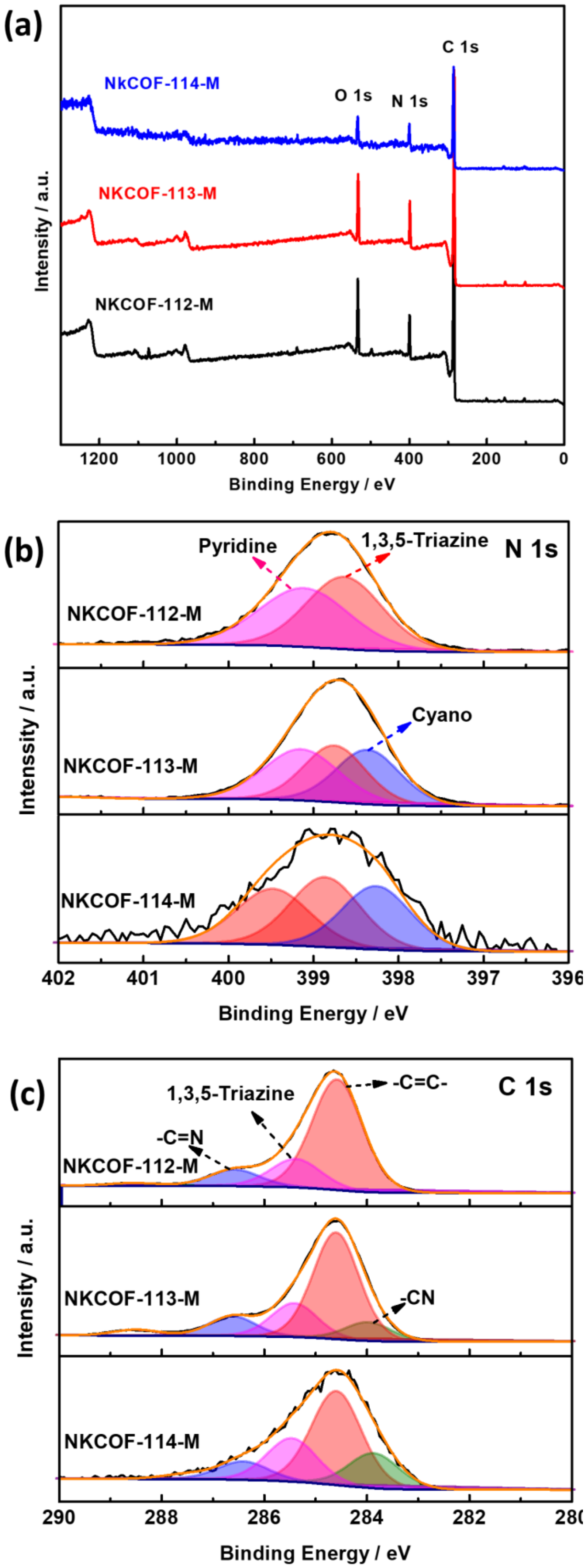

Figure S14. (a) Full scan XPS spectra and high-resolution (b) N 1s and C1s spectra of the as-synthesized COFs.

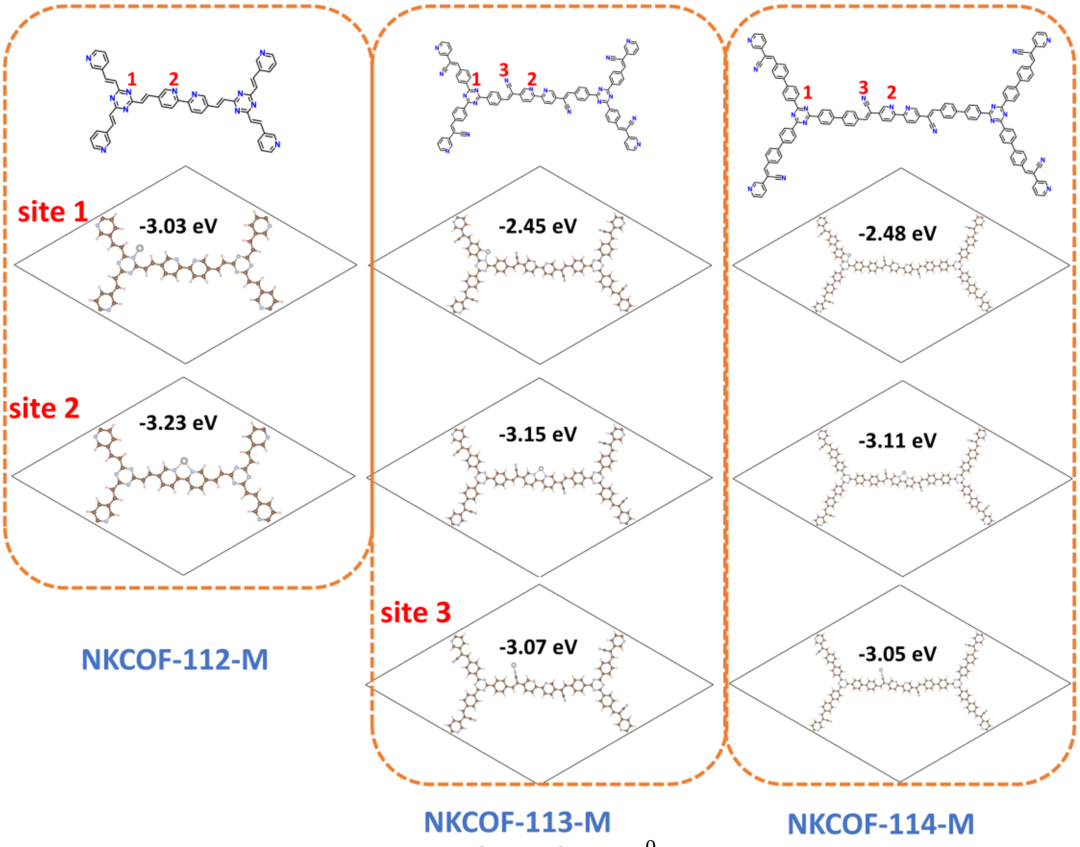

Figure S15. DFT calculated energy profiles of the Pt^0 (the white ball) adsorption at the N sites. site 1: TA; site 2: Bpy; site 3 Cyano.

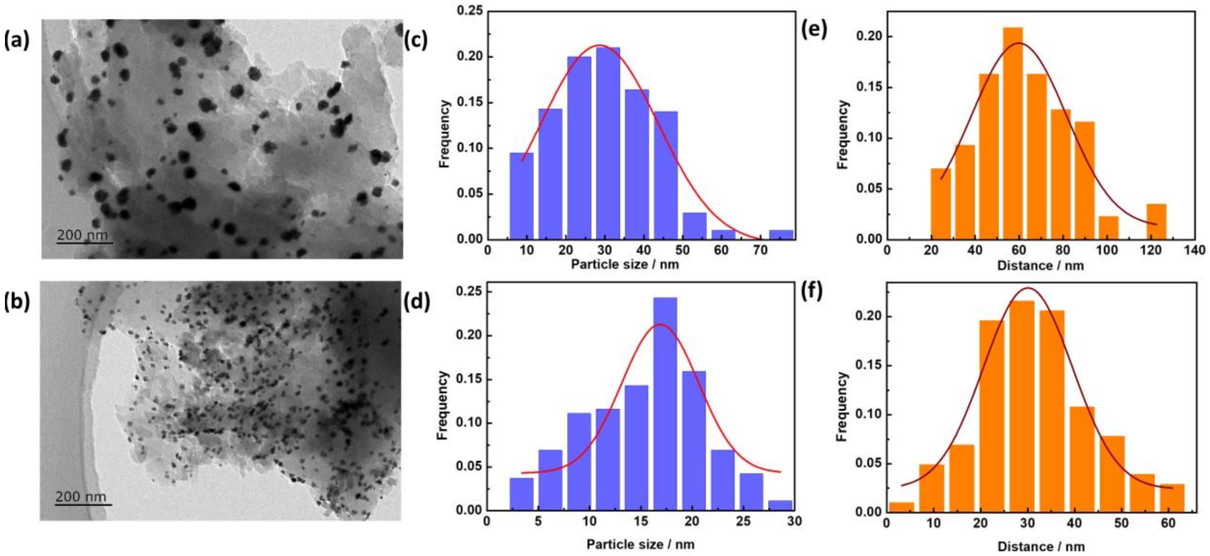

Figure S16. The TEM images of (a) Pt@NKCOF-112-M, (b) Pt@NKCOF-114-M, The Pt nanoparticle size distribution on the surface of (c) NKCOF-112-M, (d) NKCOF-114-M; Statistical distances between centers of neighboring Pt nanoparticles on the surface of (e) NKCOF-112-M, (f) NKCOF-114-M.

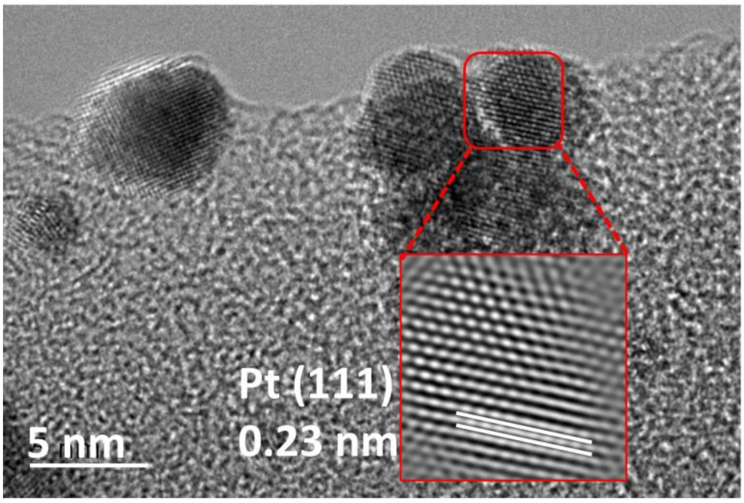

Figure S17. The HRTEM image of NKCOF-113-M.

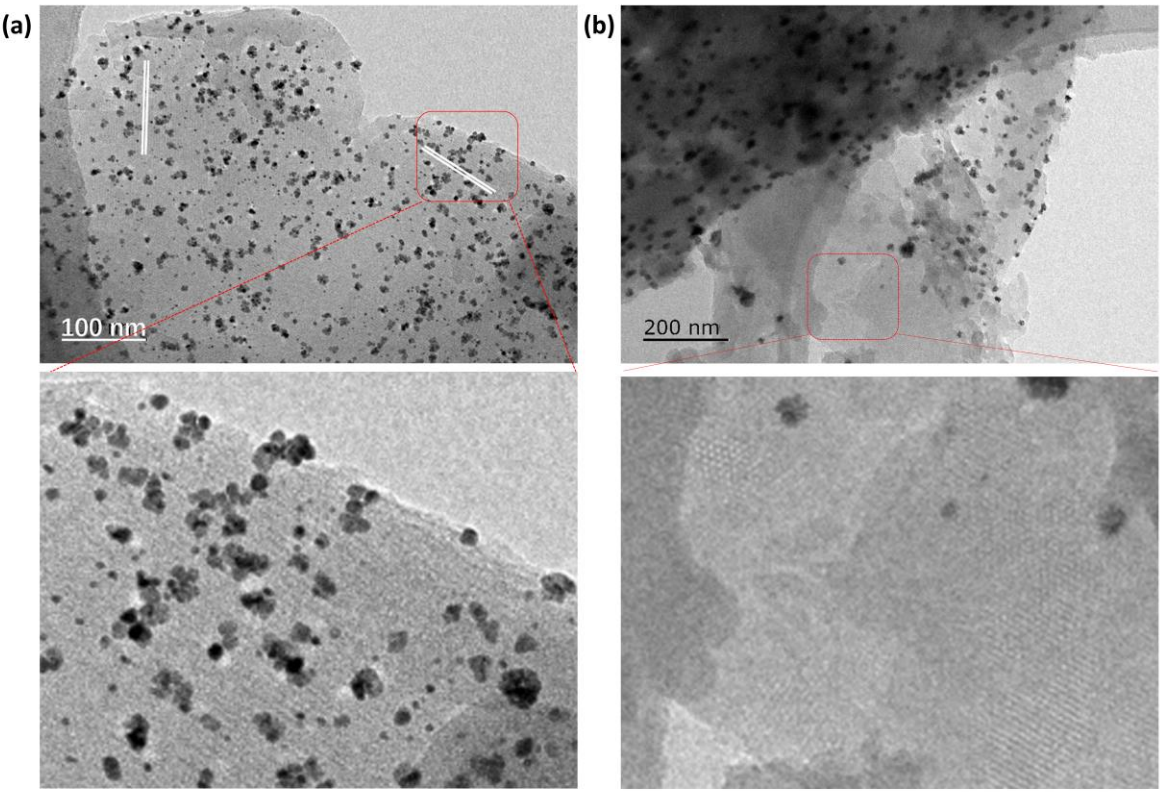

Figure S18. The TEM image of the (a) Pt@NKCOF-113-M and (b) Pt@NKCOF-114-M.

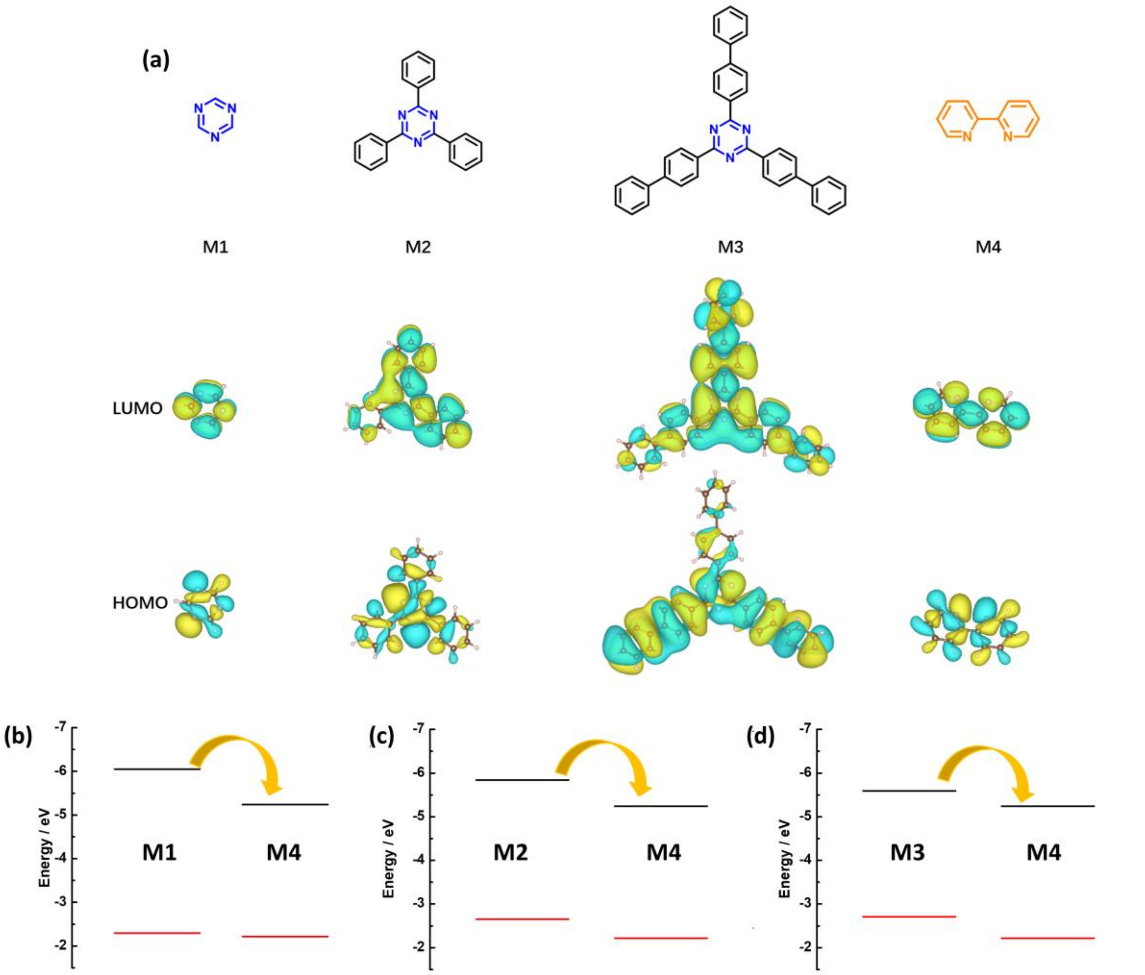

Figure S19. (a) The frontier orbital distribution of the model molecules and the possible energy/electron transfer between the model molecules according to their energy levels in the COF skeleton: (b) NKCOF-112-M, (b) NKCOF-113-M, (c) NKCOF-114-M.

Table S1. Apparent quantum Yield (AQY) of the COF photocatalysts for H_2 evolution.

**COFs**	**HER (mmol h^{-1} g^{-1})**	**AQY**	**Conditions**	**Reference**
NKCOF-113-M	***13.1***	***56.2% (475 nm)***	***TEOA λ > 420 nm***	***This work***
CYANO-CON	134.2	82.6% (450 nm)	AA λ > 420 nm	Nat Commun, **2022**, 13 (1): 2357
CN-COF	10.1	20.7% (425 nm)	TEOA λ > 420 nm	Chem. Commun., **2019**, 55 (41): 5829-5832
TpPa-Cl_2	--	17% (400 nm)	SA λ > 420 nm	Chem.Eng. J., **2021**, 419
WO_3@TpPa-1-COF/rGO (30%)	16.65	12.08% (450 nm)	SA λ > 420 nm	Chem**.** Eng. J., **2022**, 431

r-CTF	10.25	11.3% (420 nm)	TEOA λ > 420 nm	Angew.Chem. Int.Ed. **2021**, 60,25381–25390
PEG@BT-COF	11.4	11.2% (420 nm)	AA λ > 420 nm	Nat. Commun., 2021, 12 (1): 3934.
ATNT-4	14.2	9.75% (500 nm)	AA λ > 420 nm	J. Am. Chem. Soc., **2020**, 142 (10): 4862-4871
Py-ClTP-BT-COF	8.9	8.45% (420 nm)	AA λ > 420 nm	Angew. Chem. Int. Ed., **2020**, 59, 2.
Pt-PY-DHBD-COF	71.16	8.4% (420 nm)	AA λ > 420 nm	Nat. Commun, **2022**, 13 (1): 1355.
TiO_2-TpPa-1-COF	11.19	7.6% (420 nm)	SA λ > 420 nm	Appl. Catal. B: Environ., **2020**, 266: 118586
CTF-HUST-A1	9.2	7.4% (420 nm)	TEOA λ > 420 nm	Angew. Chem. Int. Ed., **2020**, 59, 6007.
$Pd_{0.033}$/TPTTA/SiO_2	150	7.3% (420 nm)	AA λ > 420 nm	ACS Appl. Mater. Interfaces 2022, 14, 6885−6893
COF-alkene	2.33	6.7% (420 nm)	TEOA λ > 420 nm	Adv. Sci., 2020, 7 (12): 1902988
RC-COF-1	~28	6.39% (420 nm)	AA λ > 420 nm	Nature, **2022**, 604 (7904): 72-79.
g-$C_{40}N_3$-COF	4.12	4.84% (420 nm)	TEOA λ > 420 nm	Nat. Commun., **2019**, 10:2467
sonoCOF-3	16.6	3.71% (420 nm)	AA λ > 420 nm	Nat. Synth., **2022**, 1 (1): 87-95
FS-COF	10.1	3.2% (420 nm)	AA λ > 420 nm	Nat. Chem., **2018**, 10, 1180
NKCOF-108	11.6	2.96% (520 nm)	AA λ > 420 nm	ACS Catal., 2021, 11 (4): 2098-2107
P-CTF	6.59	2.11%	TEOA	Chem. Mater.,

		(420 nm)	λ > 420 nm	2022, 34 (4): 1481-1490
COF-BPDA	3.23	2.1% (435 nm)	AA λ > 400 nm	J Am Chem Soc, 2022, 144 (8): 3653-3659
BTH-1	10.5	1.569% (420 nm)	AA λ > 420 nm	Nat. Commun., 2022, 13 (1): 100
TZ-COF-4	4.3	1.3% (420 nm)	AA λ > 420 nm	J. Am. Chem. Soc. **2020**, 142, 11131
ODA-COF	2.6	0.42% (420)	TEOA λ > 420 nm	Angew. Chem. Int. Ed., **2022**, 61 (10): e202115655

AA：Ascorbic acid; TEOA: Triethanolamine; SA: Sodium ascorbate

Table S2. Fractional atomic coordinates of the NKCOF-112-M unit cell.

**Space group**	P-6		
**Calculated unit cell**	a = 30.1062, b = 30.1062, c =3.4475<br>α=β= 90°, γ=120°		
**Atom**	x	y	z
C1	0.13631	0.59063	0.5
C2	0.10773	0.61577	0.5
C3	0.05424	0.58793	0.5
C4	0.02869	0.53402	0.5
N5	0.05723	0.51042	0.5
C6	0.10933	0.53667	0.5
C7	-0.02811	0.5013	0.5
N8	-0.04857	0.44947	0.5
C9	-0.09992	0.41624	0.5
C10	-0.13462	0.43444	0.5
C11	-0.1145	0.48786	0.5
C12	-0.06156	0.52122	0.5
C13	0.19321	0.62225	0.5
C14	0.22516	0.6033	0.5
C15	0.28134	0.63659	0.5
C16	-0.19043	0.39647	0.5
C17	-0.22774	0.40849	0.5
C18	-0.28252	0.36911	0.5
N19	0.30344	0.68852	0.5
N20	-0.31833	0.38392	0.5
H21	0.12689	0.65727	0.5
H22	0.03404	0.60907	0.5
H23	0.12877	0.51479	0.5
H24	-0.1136	0.37545	0.5

H25	-0.13889	0.50461	0.5
H26	-0.04788	0.56186	0.5
H27	0.20952	0.66351	0.5
H28	0.21071	0.5625	0.5
H29	-0.20121	0.35631	0.5
H30	-0.21889	0.44792	0.5

Table S3. Fractional atomic coordinates of the NKCOF-113-M unit cell.

**Space group**	P-6		
**Calculated unit cell**	a = 45.3935, b = 45.3935, c =3.4485<br>α=β= 90°, γ=120°		
**Atom**	x	y	z
C1	0.09031	0.57463	0.5
C2	0.06795	0.58782	0.5
C3	0.03275	0.56648	0.5
C4	0.01898	0.53111	0.5
N5	0.04064	0.51862	0.5
C6	0.07502	0.53876	0.5
C7	0.98181	0.50646	0.5
N8	0.97144	0.47273	0.5
C9	0.93817	0.44811	0.5
C10	0.91205	0.45646	0.5
C11	0.92258	0.49153	0.5
C12	0.957	0.51625	0.5
C13	0.12848	0.59855	0.5
C14	0.15092	0.58696	0.5
C15	0.18919	0.60589	0.5
C16	0.87506	0.42851	0.5
C17	0.84913	0.4354	0.5
C18	0.81121	0.41198	0.5
C19	0.20503	0.58594	0.5
C20	0.24038	0.60079	0.5
C21	0.26125	0.63625	0.5
C22	0.24587	0.65644	0.5
C23	0.21047	0.64159	0.5
C24	0.791	0.42754	0.5
C25	0.75554	0.40831	0.5
C26	0.73894	0.37268	0.5
C27	0.7587	0.35684	0.5
C28	0.79422	0.37608	0.5
H29	0.07735	0.6149	0.5
H30	0.01706	0.57812	0.5
H31	0.08946	0.52588	0.5
H32	0.93251	0.42199	0.5
H33	0.90504	0.50094	0.5
H34	0.9635	0.54258	0.5
C35	0.13999	0.63453	0.5
H36	0.14069	0.55993	0.5

C37	0.86857	0.39395	0.5
H38	0.8557	0.46163	0.5
H39	0.25129	0.58434	0.5
H40	0.26121	0.68392	0.5
H41	0.74118	0.42148	0.5
H42	0.74673	0.32937	0.5
C43	0.29879	0.65209	0.5
C44	0.7013	0.35219	0.5
N45	0.31887	0.68648	0.5
N46	0.68232	0.3677	0.5
H47	0.18995	0.55843	0.5
H48	0.20108	0.65898	0.5
H49	0.80269	0.455	0.5
H50	0.8071	0.36181	0.5
N51	0.14843	0.66319	0.5
N52	0.86419	0.36652	0.5

Table S4. Fractional atomic coordinates of the NKCOF-114-M unit cell.

**Space group**	P-6		
**Calculated unit cell**	a = 60.5891, b = 60.5891, c =3.4482<br>α=β= 90°, γ=120°		
**Atom**	x	y	z
C1	0.06762	0.56129	0.5
C2	0.05076	0.57105	0.5
C3	0.02441	0.55495	0.5
C4	0.01421	0.52847	0.5
N5	0.03054	0.51923	0.5
C6	0.05629	0.53442	0.5
C7	-0.01361	0.50989	0.5
N8	-0.02125	0.48465	0.5
C9	-0.04615	0.46612	0.5
C10	-0.06581	0.47224	0.5
C11	-0.05804	0.49848	0.5
C12	-0.03229	0.5171	0.5
C13	0.09619	0.57932	0.5
C14	0.11311	0.57076	0.5
C15	0.14179	0.58507	0.5
C16	-0.09348	0.45121	0.5
C17	-0.11299	0.45624	0.5
C18	-0.14139	0.43858	0.5
C19	0.15379	0.57034	0.5
C20	0.18028	0.58155	0.5
C21	0.19631	0.60829	0.5
C22	0.18415	0.62302	0.5
C23	0.1576	0.61173	0.5
C24	-0.15663	0.45002	0.5
C25	-0.18319	0.43554	0.5
C26	-0.19602	0.40863	0.5

C27	-0.18061	0.39721	0.5
C28	-0.15401	0.41178	0.5
H29	0.05771	0.59132	0.5
H30	0.01257	0.56358	0.5
H31	0.0672	0.52486	0.5
H32	-0.05029	0.44658	0.5
H33	-0.07126	0.50545	0.5
H34	-0.02752	0.5368	0.5
C35	0.10469	0.60625	0.5
H36	0.10554	0.55053	0.5
C37	-0.0982	0.42536	0.5
H38	-0.10816	0.47586	0.5
H39	0.18753	0.56857	0.5
H40	0.1946	0.64351	0.5
H41	-0.19307	0.44616	0.5
H42	-0.18848	0.37689	0.5
C43	0.22512	0.62057	0.5
C44	-0.22489	0.3928	0.5
C45	0.24116	0.64738	0.5
C46	0.26768	0.65864	0.5
C47	0.2793	0.64365	0.5
C48	0.26377	0.61709	0.5
C49	0.23725	0.60575	0.5
C50	-0.23773	0.36582	0.5
C51	-0.26431	0.35128	0.5
C52	-0.27919	0.36302	0.5
C53	-0.26686	0.38968	0.5
C54	-0.24029	0.40429	0.5
H55	0.14256	0.54972	0.5
H56	0.15043	0.62464	0.5
H57	-0.14795	0.47059	0.5
H58	-0.14422	0.40121	0.5
C59	0.30744	0.65564	0.5
C60	-0.30739	0.34756	0.5
N61	0.31859	0.64098	0.5
N62	-0.32171	0.35908	0.5
H63	0.2339	0.66035	0.5
H64	0.27908	0.67924	0.5
H65	0.27209	0.60489	0.5
H66	0.22683	0.58526	0.5
H67	-0.22784	0.35521	0.5
H68	-0.27317	1.33071	0.5
H69	-0.27775	0.39942	0.5
H70	-0.23246	0.42461	0.5
N71	0.11091	0.6277	0.5
N72	-0.10139	0.40485	0.5

Table S5. The pore structure of the reported olefin-linked COFs

COFs	Shape of the pore	Pore size (nm)	References
NKCOF-112-M<br>***NKCOF-113-M***<br>***NKCOF-114-M***	***Hexagonal***<br>***Hexagonal***<br>***Hexagonal***	***2.2***<br>***3.8***<br>***5.6***	*This work*
Sp^2-COF	Quadrilateral	1.9	Science, **2017**, 357, 673
Sp^2-COF-2<br>Sp^2-COF-3	Quadrilateral	2.4<br>2.7	Nat. Commun. **2018**, 9:4143
Sp^2-COF-4<br>Sp^2-COF-5	Hexagonal<br>Hexagonal and Triangle	3.2<br>3.6 and 1.4	Angew. Chem. Int. Ed. **2020**, 59, 12162
Sp^2-COF-6<br>Sp^2-COF-7<br>Sp^2-COF-8<br>Sp^2-COF-9<br>Sp^2-COF-10<br>Sp^2-COF-11<br>Sp^2-COF-12<br>Sp^2-COF-13<br>Sp^2-COF-14	Hexagonal<br>Hexagonal<br>Hexagonal<br>Hexagonal<br>Hexagonal<br>Hexagonal<br>Hexagonal<br>Hexagonal<br>Quadrilateral	1.9<br>1.7<br>1.7<br>1.8<br>1.7<br>2.5<br>2.5<br>2.0<br>2.0	Angew. Chem. Int. Ed. **2022**, 61, e2021150
2DPPV	Hexagonal	1.6	Polym. Chem., **2016**, 7, 4176
g-$C_{40}N_3$-COF<br>g-$C_{31}N_3$-COF<br>g-$C_{37}N_3$-COF	Hexagonal	3.2<br>2.4<br>1.6	Nat. Commun. **2019**, 10:2467
g-$C_{34}N_6$-COF	Hexagonal	1.6	Angew. Chem. Int. Ed. **2019**, 58,12065
COF-p-3Ph,<br>COF-p-2Ph<br>COF-m-3Ph	Hexagonal	3.0<br>2.2<br>1.7	J. Am. Chem. Soc. **2020**, 142, 11893
COF-DFB<br>COF-BPDA	Quadrilateral	1.4<br>1.7	J. Am. Chem. Soc. **2022**, 144, 3653
Por-sp2c-COF	Quadrilateral	1.8	Angew. Chem. Int. Ed. **2019**, 131, 6496
COF1<br>COF2<br>COF3	Hexagonal	1.1	Angew. Chem. Int. Ed. **2019**, 58,13753
V-COF-1<br>V-COF-2	Hexagonal	1.7<br>0.8	Angew. Chem. Int. Ed. **2019**, 58, 14865
P2PV<br>P3PcB<br>P2NV<br>P3NcB	Hexagonal	1.1	J. Am. Chem. Soc. **2020**, 142, 8862
DBOV-COF	Hexagonal	1.9	Angew. Chem.Int. Ed. **2022**, 61, e2021140
V-2D-COF-1<br>V-2D-COF-2<br>V-2D-COF-3<br>V-2D-COF-4	Hexagonal<br>Hexagonal<br>Hexagonal<br>Triangle	2.3~3.2<br>2.3~3.2<br>3.2~4.2<br>1.3 and 1.8	Angew. Chem. Int. Ed. **2022**, *61*, e202202492
Bpy-sp^2c-COF	Quadrilateral	2.4	Chem. Sci. **2020**, 11, 543
TP-COF	Hexagonal	2.2	Angew. Chem. Int. Ed. **2019**,

			58,5376
BTH-1<br>BTH-2<br>BTH-3	Hexagonal	~2.5	Nat. Commun. **2022**, 13: 100
NKCOF-10	Hexagonal	1.8	Nat. Commun., **2021**, 12: 1982
NKCOF-12	Hexagonal	0.6	Sci. China. Chem., **2022**, 65, 1173
NKCOF-113	Hexagonal	3.9	*Angew. Chem. Int. Ed.* **2022**, e202200261

Table S6. The PL decay lifetime of the COFs.

	Decay lifetime (ns)			
**Sample**	τ_1	τ_2	τ_3	τ_{Ave}
**NKCOF-112-M**	1.02	2.71	9.23	2.5
**NKCOF-113-M**	0.83	3.03	8.87	2.8
**NKCOF-114-M**	0.66	2.43	9.12	2.2

Table S7. The measurements of the Hall effect for the COFs

			Hall effect			
Sample	**T (K)**	**σ (Ω·cm)**	**μ (cm^2/ V·S)**	**n (cm^{-3})**	**R_H (cm^{-3}/C)**	**f-factor**
NKCOF-112-M	300	$4.21 \cdot 10^3$	0.12	$1.24 \cdot 10^{16}$	-0.504	0.687
NKCOF-113-M	300	$3.19 \cdot 10^3$	0.13	$1.27 \cdot 10^{16}$	-0.496	0.691
NKCOF-114-M	300	$2.16 \cdot 10^3$	0.137	$1.30 \cdot 10^{16}$	-0.309	0.670

T: Temperature; σ: Resistivity; μ: Charge Mobility; n: Carrier concentration; R_H: Hall coefficient

Supplementary References

[1] a)G. Kresse, J. Furthmüller, *Comp. Mater. Sci.* **1996**, 6, 15; b)G. Kresse, J. Furthmuller, *Phys. Rev. B Condens Matter.* **1996**, 54, 11169.

[2] a)P. E. Blochl, *Phys. Rev. B Condens Matter.* **1994**, 50, 17953; b)G. Kresse, D. Joubert, *Phys. Rev. B* **1999**, 59, 1758.

[3] J. P. Perdew, K. Burke, M. Ernzerhof, *Phys. Rev. Lett.* **1996**, 77, 3865.

[4] L. Y. Liao, X. R. Kong, X. F. Duan, *J. Org. Chem.* **2014**, 79, 777.
